# Triglyceride-glucose index, CKM stage, and absolute benefit of intensive blood pressure control: a *post hoc* analysis of SPRINT

**DOI:** 10.3389/fnut.2026.1859944

**Published:** 2026-07-16

**Authors:** Na Li, Quan Yuan, Zhixin Yang, Wenli Cheng

**Affiliations:** 1 The Second Department of Healthcare, China-Japan Friendship Hospital, Beijing, China; 2 Department of Information Systems and Operations Management, Ball State University, Muncie, IN, United States; 3 Department of Mathematical Sciences, Ball State University, Muncie, IN, United States; 4 Centre of Hypertension, Beijing Anzhen Hospital, Capital Medical University, Beijing, China

**Keywords:** absolute risk reduction, cardiovascular-kidney-metabolic (CKM), intensive blood pressure control, serious adverse events, SPRINT, triglyceride-glucose index

## Abstract

**Background:**

Intensive blood pressure control reduces cardiovascular events and all-cause mortality in SPRINT, but absolute benefit varies by baseline characteristics. The triglyceride-glucose (TyG) index may provide additional descriptive information within the cardiovascular-kidney-metabolic (CKM) staging framework.

**Methods:**

We performed a *post hoc* analysis of SPRINT. CKM stages 2–4 were classified from baseline characteristics by programmatically applying the AHA CKM staging criteria. Participants whose CKM stage could not be determined because PREVENT-based risk staging inputs were missing or outside the PREVENT/preventR calculator-supported domain were excluded from analyses requiring CKM stage assignment. Cox models adjusted for age, sex, baseline systolic blood pressure, and estimated glomerular filtration rate evaluated the SPRINT primary composite outcome and all-cause death. Formal interaction tests assessed whether intensive versus standard blood pressure treatment effects varied by CKM stage, TyG, or their combination. Three-year absolute risk reduction (ARR) was estimated using Cox-standardized survival prediction. Exploratory safety-adjusted net-benefit analyses incorporated serious adverse events, acute kidney injury, and hypotension.

**Results:**

Among 9,323 TyG-calculable participants, 8,036 were included in the main complete-case cohort; 1,282 remained unclassified by the CKM algorithm. Intensive blood pressure treatment reduced the primary composite outcome (HR 0.73, 95% CI 0.63–0.86) and all-cause death (HR 0.81, 95% CI 0.67–0.98). Formal testing showed nominal evidence that the treatment effect on all-cause death varied by TyG (*P* = 0.025), but not that this TyG-related effect differed across CKM stages (treatment × CKM stage × TyG interaction, *P* = 0.775). Cox-standardized ARR estimates suggested the largest absolute mortality benefit among participants with CKM stage 4 and high TyG (ARR 3.4 percentage points, 95% CI 1.3–5.6). Fasting-only and exploratory safety-adjusted analyses were directionally similar.

**Conclusion:**

In SPRINT, TyG and CKM stage jointly described exploratory gradients in absolute benefit from intensive blood pressure treatment, particularly for all-cause death. Because analyses requiring CKM stage assignment excluded unclassified participants and formal interaction evidence was limited, these findings should be interpreted as hypothesis-generating absolute-risk stratification rather than as a treatment-selection rule.

## 1 Introduction

The Systolic Blood Pressure Intervention Trial (SPRINT) demonstrated that intensive systolic blood pressure control reduced major cardiovascular events and all-cause mortality in high-risk adults without diabetes (1, 2). However, the absolute benefit of intensive blood pressure lowering is not uniform across all participants, and identifying subgroups with greater absolute benefit, while also accounting for treatment-related harms, remains clinically important (3).

The cardiovascular-kidney-metabolic (CKM) framework integrates cardiovascular, kidney, and metabolic risk into a unified staging system (4). Recent analyses have applied CKM staging and risk-stratification approaches to SPRINT and suggested that treatment benefit may vary most clearly on the absolute risk scale (3, 5). Given its prior associations with cardiovascular, kidney, and mortality outcomes, TyG was evaluated as an exploratory metabolic marker within CKM stages. The triglyceride-glucose (TyG) index, calculated from triglycerides and glucose, is an inexpensive surrogate of insulin resistance (6). Prior studies have linked TyG to atherosclerotic cardiovascular disease, incident cardiovascular disease, chronic kidney disease, and mortality (7–17).

## 2 Materials and methods

### 2.1 Study design and data source

This was a *post hoc* analysis of SPRINT using trial data obtained from the NHLBI BioLINCC repository (18). SPRINT enrolled hypertensive adults at increased cardiovascular risk and excluded participants with diabetes mellitus or prior stroke (1, 2). The present analysis used baseline variables, adjudicated efficacy outcomes, and trial safety outcomes available in the SPRINT data files.

### 2.2 CKM staging algorithm

Because CKM stage was not prospectively assigned in SPRINT, we programmatically implemented the AHA CKM framework by applying its staging criteria to baseline trial variables. This baseline-variable–based implementation followed the AHA CKM stage definitions to the extent supported by the SPRINT analytic files. Hereafter, references to “CKM stage” and “CKM staging” refer to this programmatic implementation of AHA CKM staging. The TyG-eligible cohort included 9,323 participants. The classification algorithm was hierarchical and used baseline variables only. CKM stage 4 was assigned first based on indicators of clinical cardiovascular disease, including myocardial infarction, heart attack, acute coronary syndrome, coronary revascularization, carotid disease, peripheral arterial revascularization, atrial fibrillation, angina, congestive heart failure, stroke, and peripheral vascular disease. Participants meeting any of these criteria were classified as CKM stage 4 regardless of PREVENT risk availability.

Among participants not classified as CKM stage 4, CKM stage 3 was assigned if they had very-high-risk CKD according to KDIGO risk classification, a positive cardiovascular disease history/subclinical cardiovascular disease indicator, or high predicted 10-year cardiovascular disease risk. High predicted risk was defined as PREVENT 10-year total cardiovascular disease risk greater than or equal to 20%. PREVENT risk was calculated using the base PREVENT model implemented in the preventR package (19, 20). Risk-based CKM3 classification was applied only when required PREVENT inputs were available and within the PREVENT/preventR calculator-supported input domain. Required PREVENT inputs included sex, age, total cholesterol, HDL cholesterol, systolic blood pressure, BMI, eGFR, diabetes status, current smoking status, antihypertensive medication use, and lipid-lowering therapy use. Participants with missing required PREVENT inputs or inputs outside the PREVENT/preventR calculator-supported domain were not assigned CKM3 on the basis of PREVENT risk. Optional predictors, including UACR, HbA1c, and social deprivation index, were not included in the primary PREVENT model.

Participants not meeting CKM stage 4 or non-PREVENT CKM stage 3 criteria and without a valid PREVENT estimate were left unclassified rather than being forced into CKM stage 2 or 3. This approach avoided assigning risk-based CKM stage 2 or 3 status when PREVENT-based risk staging could not be performed because required inputs were missing or outside the PREVENT/preventR calculator-supported domain. Participants not classified as CKM stage 4 or 3 and with valid PREVENT risk below 20% were classified as CKM stage 2. This algorithm classified 8,041 of 9,323 TyG-eligible participants as CKM stage 2, 3, or 4; 1,282 participants remained unclassified. Among classified TyG-eligible participants, 3,718 were assigned to CKM stage 2, 1,740 to CKM stage 3, and 2,583 to CKM stage 4. After excluding five classified participants with missing baseline eGFR, the main complete-case cohort included 8,036 participants: 3,718 with CKM stage 2, 1,740 with CKM stage 3, and 2,578 with CKM stage 4. The CKM staging algorithm, PREVENT implementation, and handling of unclassified participants are summarized in Supplementary Table 2.

### 2.3 TyG definition

The triglyceride-glucose (TyG) index was calculated as ln [triglycerides (mg/dL) × glucose (mg/dL)/2] (6). TyG was modeled primarily as a continuous variable. For clinically interpretable display, overall tertile-median TyG values were used as representative values, and tertile analyses were used for the main grouped display. Baseline fasting status for glucose and triglycerides was explicitly recorded in the SPRINT laboratory data. In the main analytic cohort, 7,526 participants had both fasting glucose and fasting triglyceride measurements, 348 had non-fasting glucose and triglyceride measurements, and 162 had unknown fasting status. Because TyG is conventionally defined using fasting triglycerides and fasting glucose, a fasting-only sensitivity analysis was performed among participants with both fasting glucose and fasting triglyceride measurements.

### 2.4 Outcomes

The primary efficacy outcome was the SPRINT primary composite endpoint, defined as myocardial infarction, acute coronary syndrome not resulting in myocardial infarction, stroke, acute decompensated heart failure, or cardiovascular death (1, 2). The key secondary outcome was all-cause death. Exploratory safety analyses evaluated global SAE, acute kidney injury SAE, and hypotension SAE, each modeled as a time-to-event outcome using the corresponding event and time variables from the SPRINT safety data (1, 2).

### 2.5 Statistical analysis

Adjusted Cox proportional hazards models were fit for the primary composite outcome and all-cause death. The main adjusted model included randomized BP-treatment assignment, CKM stage, TyG, age, sex, baseline systolic blood pressure, and eGFR. As a sensitivity analysis, we also fit expanded-adjustment complete-case Cox models including race/ethnicity indicators, BMI, current smoking, baseline BP medication count, total cholesterol, HDL cholesterol, LDL cholesterol, UACR, and aspirin use. Sex was included as a binary baseline covariate using the female indicator available in the SPRINT baseline data. Formal likelihood-ratio interaction tests evaluated intensive BP treatment × CKM stage, intensive BP treatment × TyG, intensive BP treatment × CKM stage × TyG, and the joint evidence for intensive-treatment effect modification by CKM stage or TyG. Proportional-hazards diagnostics were examined and are summarized in Supplementary Table 5.

Three-year ARR was estimated using Cox-standardized survival prediction. For each CKM/TyG stratum, the fitted Cox model was used to predict standardized 3-year risks under two counterfactual strategies: standard BP treatment and intensive BP treatment. ARR was defined as risk under standard BP treatment minus risk under intensive BP treatment; positive values therefore favor intensive BP treatment. Percentile bootstrap 95% confidence intervals were generated with 300 bootstrap samples. NNT values were displayed only when ARR was positive, the 95% confidence interval excluded zero, and ARR was at least 0.5 percentage points; otherwise, NNT was not shown.

Exploratory safety-adjusted net-benefit analyses combined efficacy ARR with harm increases from Cox-standardized safety models. Harm increase was defined as the predicted 3-year harm risk under intensive BP treatment minus the corresponding risk under standard BP treatment. Simplified net benefit was defined as ARR minus a harm-weighted increase in SAE risk. Harm weights of 0.5 and 1.0 were used as sensitivity parameters. Additional supportive analyses used AKI and hypotension as harm components. These analyses were considered exploratory because the harm weights were not validated utility weights and global SAE includes heterogeneous events.

## 3 Results

### 3.1 Cohort flow and baseline characteristics

Among 9,361 SPRINT participants with available baseline data, 9,323 had calculable TyG and 38 did not. Among participants with calculable TyG, 8,041 were classified as CKM stage 2, 3, or 4, whereas 1,282 remained unclassified because PREVENT-based risk staging could not be performed. Specifically, 648 age-eligible participants had complete required PREVENT inputs but at least one non-age PREVENT input outside the PREVENT/preventR calculator-supported domain, resulting in no valid PREVENT output row; 583 were outside the 30–79-year PREVENT age domain; 42 had missing BMI; and nine had missing eGFR. Among the 648 age-eligible participants with no valid PREVENT output, the most common out-of-domain input was BMI outside 18.5–39.9, followed by total cholesterol, systolic blood pressure, HDL cholesterol, or eGFR outside the supported ranges. After excluding five classified participants with missing baseline eGFR, the main complete-case analytic cohort included 8,036 participants. The fasting-only sensitivity cohort included 7,526 participants with both fasting glucose and fasting triglyceride measurements. Detailed cohort flow and unclassified-participant reasons are provided in Supplementary Table 1. Therefore, estimates by CKM stage should be interpreted as conditional on successful CKM stage assignment rather than as estimates for the entire TyG-calculable SPRINT cohort.

Baseline characteristics of the main analytic cohort are shown in Table 1. The cohort included 3,718 participants with CKM stage 2, 1,740 with CKM stage 3, and 2,578 with CKM stage 4. The cohort included 2,727 female participants (33.9%). Randomization to intensive BP treatment was balanced overall and across CKM stages, with 4,027 participants (50.1%) assigned to intensive BP treatment. Baseline risk increased across CKM stages: primary composite events occurred in 129 participants (3.5%) with CKM stage 2, 134 (7.7%) with CKM stage 3, and 368 (14.3%) with CKM stage 4; all-cause death occurred in 79 (2.1%), 122 (7.0%), and 219 (8.5%), respectively.

**TABLE 1:** Baseline characteristics of the main analytic cohort by cardiovascular-kidney-metabolic (CKM) stage.

Characteristic	Overall	CKM stage 2	CKM stage 3	CKM stage 4	*P*-value
Age, years	67.1 ± 8.8	62.3 ± 6.1	72.5 ± 7.2	70.5 ± 9.4	<0.001
TyG index	8.60 ± 0.54	8.62 ± 0.54	8.58 ± 0.53	8.57 ± 0.53	<0.001
Baseline systolic BP, mmHg	139.1 ± 15.0	138.1 ± 14.1	141.8 ± 15.1	138.8 ± 15.9	<0.001
Baseline diastolic BP, mmHg	78.2 ± 11.7	81.4 ± 10.4	76.0 ± 11.7	75.0 ± 12.1	<0.001
eGFR, mL/min/1.73 m^2^	71.6 ± 20.4	78.6 ± 17.8	62.0 ± 20.3	68.0 ± 20.6	<0.001
Urinary albumin-to-creatinine ratio, mg/g	9.2 (5.5, 21.0)	7.6 (5.0, 14.0)	12.2 (6.6, 37.1)	11.4 (6.1, 28.9)	<0.001
Triglycerides, mg/dL	108.0 (78.0, 152.0)	110.0 (79.0, 157.0)	106.0 (76.0, 147.0)	105.0 (76.0, 146.0)	<0.001
Glucose, mg/dL	98.9 ± 13.5	98.6 ± 13.5	98.5 ± 13.0	99.5 ± 14.0	0.002
BMI, kg/m^2^	30.0 ± 5.0	29.8 ± 4.5	29.8 ± 5.3	30.5 ± 5.9	0.012
Number of baseline BP medications	1.8 ± 1.0	1.6 ± 1.0	1.8 ± 1.0	2.1 ± 1.0	<0.001
Female	2727 (33.9%)	1400 (37.7%)	612 (35.2%)	715 (27.7%)	<0.001
Intensive BP treatment	4027 (50.1%)	1870 (50.3%)	842 (48.4%)	1315 (51.0%)	0.230
White race	5232 (65.1%)	2133 (57.4%)	1193 (68.6%)	1906 (73.9%)	<0.001
Black race	2533 (31.5%)	1460 (39.3%)	479 (27.5%)	594 (23.0%)	<0.001
Asian race	72 (0.9%)	22 (0.6%)	27 (1.6%)	23 (0.9%)	0.002
Other race	198 (2.5%)	97 (2.6%)	44 (2.5%)	57 (2.2%)	0.594
Hispanic ethnicity	862 (10.7%)	511 (13.7%)	158 (9.1%)	193 (7.5%)	<0.001
Current smoker	1175 (14.6%)	652 (17.5%)	185 (10.6%)	338 (13.1%)	<0.001

Values are mean ± SD, median (interquartile range), or *n* (%). *P*-values compare cardiovascular-kidney-metabolic (CKM) stages 2–4.

### 3.2 Main treatment effects and formal interaction tests

In the main adjusted Cox model, intensive BP treatment was associated with lower risk of the primary composite outcome (HR 0.73, 95% CI 0.63–0.86; *P* < 0.001) and all-cause death (HR 0.81, 95% CI 0.67–0.98; *P* = 0.031) compared with standard BP treatment. Formal interaction testing did not support CKM-specific three-way effect modification by intensive BP treatment. For the primary composite outcome, intensive BP treatment × CKM stage (*P* = 0.475), intensive BP treatment × TyG (*P* = 0.491), intensive BP treatment × CKM stage × TyG (*P* = 0.277), and the joint interaction test (*P* = 0.482) were not significant. For all-cause death, the intensive BP treatment × TyG interaction was nominally significant (*P* = 0.025), whereas intensive BP treatment × CKM stage (*P* = 0.230), intensive BP treatment × CKM stage × TyG (*P* = 0.775), and the joint interaction test (*P* = 0.116) were not significant. Fasting-only sensitivity analyses yielded similar overall treatment-effect estimates, with HR 0.75 (95% CI 0.63–0.88; *P* < 0.001) for the primary composite outcome and HR 0.80 (95% CI 0.65–0.97; *P* = 0.026) for all-cause death. Therefore, ARR analyses were interpreted as exploratory absolute-benefit patterns rather than definitive evidence of CKM-specific intensive-treatment effect heterogeneity. Expanded-adjustment complete-case sensitivity analyses produced directionally consistent main intensive BP treatment HRs, although CKM/TyG-specific ARR estimates were attenuated after expanded adjustment and complete-case restriction (Supplementary Table 10). The corresponding main treatment effects and formal interaction tests are summarized in Table 2.

**TABLE 2:** Main effects and formal interaction tests for intensive BP treatment in the main analytic cohort.

(A) Main adjusted treatment effects					
**Outcome**	**Intensive BP treatment vs. standard BP treatment HR (95% CI)**			***P*-value**	
All-cause death	0.81 (0.67, 0.98)			0.031	
Primary composite outcome	0.73 (0.63, 0.86)			<0.001	
(B) Formal interaction tests					
**Outcome**	**Interaction test**	**Chi-square**	**df**	***P*-value**	**Interpretation**
All-cause death	Intensive BP treatment × CKM	2.94	2	0.23	No nominal evidence of interaction
All-cause death	Intensive BP treatment × TyG	5.04	1	0.025	Nominal evidence of interaction
All-cause death	Intensive BP treatment × CKM × TyG	0.51	2	0.775	No nominal evidence of interaction
All-cause death	Any intensive-treatment effect modification by CKM stage or TyG	8.84	5	0.116	No nominal evidence of interaction
Primary composite outcome	Intensive BP treatment × CKM	1.49	2	0.475	No nominal evidence of interaction
Primary composite outcome	Intensive BP treatment × TyG	0.47	1	0.491	No nominal evidence of interaction
Primary composite outcome	Intensive BP treatment × CKM × TyG	2.57	2	0.277	No nominal evidence of interaction
Primary composite outcome	Any intensive-treatment effect modification by CKM stage or TyG	4.48	5	0.482	No nominal evidence of interaction

Full treatment-effect estimates, interaction-test results, and proportional-hazards diagnostics are provided in Supplementary Table 5. Schoenfeld residual diagnostics did not show nominal proportional-hazards departure for the randomized intensive BP treatment contrast in the main or fasting-only Cox models; selected covariate-level departures are reported in Supplementary Table 5. Intensive BP treatment denotes randomized assignment to intensive systolic blood pressure control versus standard control. Cardiovascular-kidney-metabolic (CKM) stage was modeled as a three-level categorical variable with CKM stage 2 as the reference; CKM interaction tests are global likelihood-ratio tests.

### 3.3 Cox-standardized 3-year ARR

**FIGURE 1:**
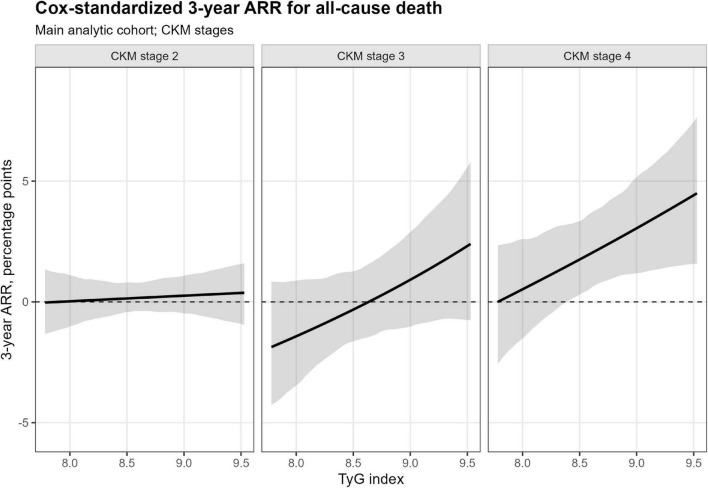
Cox-standardized 3-year absolute risk reduction (ARR) for all-cause death across continuous triglyceride-glucose (TyG) by cardiovascular-kidney-metabolic (CKM) stage in the main analytic cohort.

Cox-standardized 3-year ARR estimates showed the clearest exploratory absolute-benefit pattern for all-cause death. Across low, middle, and high TyG tertile-median values, death ARR was 0.0%, 0.2%, and 0.3% in CKM stage 2; −1.3%, −0.2%, and 1.2% in CKM stage 3; and 0.7%, 1.9%, and 3.4% in CKM stage 4. The largest estimated mortality benefit was observed in CKM stage 4 with high TyG, with ARR 3.4 percentage points (95% CI 1.3 to 5.6) (Figure 1 and Table 3).

**TABLE 3:** Cox-standardized 3-year absolute risk reduction at triglyceride-glucose (TyG) tertile-median values by cardiovascular-kidney-metabolic (CKM) stage.

Outcome	CKM stage	TyG tertile	TyG median	3-year risk, standard BP/intensive BP (%)	ARR, percentage points (95% CI)	NNT
All-cause death	CKM stage 2	T1 (low TyG)	8.08	1.3/1.3	0.0 (−0.9, 1.0)	–
All-cause death	CKM stage 2	T2 (middle TyG)	8.56	1.4/1.2	0.2 (−0.4, 0.8)	–
All-cause death	CKM stage 2	T3 (high TyG)	9.11	1.4/1.2	0.3 (−0.5, 1.2)	–
All-cause death	CKM stage 3	T1 (low TyG)	8.08	3.5/4.8	−1.3 (−3.1, 0.9)	–
All-cause death	CKM stage 3	T2 (middle TyG)	8.56	4.3/4.5	−0.2 (−1.5, 1.4)	–
All-cause death	CKM stage 3	T3 (high TyG)	9.11	5.3/4.1	1.2 (−0.9, 3.4)	–
All-cause death	CKM stage 4	T1 (low TyG)	8.08	5.7/5.0	0.7 (−1.1, 2.6)	–
All-cause death	CKM stage 4	T2 (middle TyG)	8.56	6.5/4.5	1.9 (0.5, 3.6)	53
All-cause death	CKM stage 4	T3 (high TyG)	9.11	7.4/4.1	3.4 (1.3, 5.6)	30
Primary composite outcome	CKM stage 2	T1 (low TyG)	8.08	2.7/1.7	1.1 (−0.0, 2.2)	–
Primary composite outcome	CKM stage 2	T2 (middle TyG)	8.56	3.1/1.9	1.2 (0.4, 2.1)	81
Primary composite outcome	CKM stage 2	T3 (high TyG)	9.11	3.6/2.2	1.4 (0.2, 2.7)	71
Primary composite outcome	CKM stage 3	T1 (low TyG)	8.08	4.9/5.3	−0.3 (−2.8, 2.0)	–
Primary composite outcome	CKM stage 3	T2 (middle TyG)	8.56	6.3/5.2	1.1 (−0.7, 3.1)	–
Primary composite outcome	CKM stage 3	T3 (high TyG)	9.11	8.3/5.1	3.2 (0.6, 5.9)	32
Primary composite outcome	CKM stage 4	T1 (low TyG)	8.08	10.9/8.3	2.6 (0.0, 5.3)	39
Primary composite outcome	CKM stage 4	T2 (middle TyG)	8.56	12.6/9.8	2.8 (0.8, 4.8)	36
Primary composite outcome	CKM stage 4	T3 (high TyG)	9.11	14.7/11.7	3.0 (−0.4, 6.5)	–

Full main-cohort and fasting-only Cox-standardized absolute risk reduction (ARR) estimates are provided in Supplementary Table 6. Stratum-specific intensive BP treatment HRs shown side by side with standardized 3-year risks and ARR estimates are provided in Supplementary Table 9. NNT is displayed only when ARR is positive, the 95% CI excludes zero, and ARR is at least 0.5 percentage points; otherwise, NNT is not shown.

**FIGURE 2:**
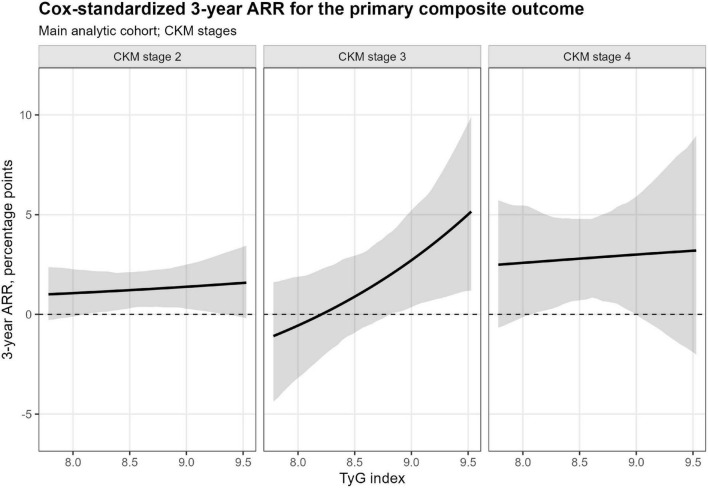
Cox-standardized 3-year absolute risk reduction (ARR) for the primary composite outcome across continuous triglyceride-glucose (TyG) by cardiovascular-kidney-metabolic (CKM) stage in the main analytic cohort.

For the primary composite outcome, ARR estimates were generally positive in CKM stage 2 and CKM stage 4 and increased across TyG tertiles in CKM stage 3, but the pattern was less consistent than for all-cause death. Across low, middle, and high TyG tertile-median values, primary-outcome ARR was 1.1%, 1.2%, and 1.4% in CKM stage 2; −0.3%, 1.1%, and 3.2% in CKM stage 3; and 2.6%, 2.8%, and 3.0% in CKM stage 4 (Figure 2 and Table 3). Several confidence intervals crossed zero, especially in CKM stage 3 and in high-TyG CKM stage 4 for the primary composite outcome. Fasting-only sensitivity analyses yielded qualitatively similar ARR patterns (Supplementary Table 6). Because formal interaction tests did not show statistically significant CKM-specific three-way intensive-treatment effect modification, these ARR estimates should be interpreted as exploratory absolute-risk descriptions rather than evidence that TyG identifies treatment responders within CKM stages. Stratum-specific intensive BP treatment HRs, standardized 3-year risks under standard and intensive BP treatment, ARR estimates, and NNT display rules are provided side by side in Supplementary Table 9.

### 3.4 Exploratory safety-adjusted net benefit

Safety analyses used event and time variables from the SPRINT safety data in the main analytic cohort. Global SAE events occurred in 2,992 participants, AKI SAE events in 272, and hypotension SAE events in 160. Intensive BP treatment was associated with higher observed AKI and hypotension event rates across most CKM strata, consistent with known SPRINT safety patterns (1, 2).

Cox-standardized harm-increase estimates showed that AKI and hypotension were more directionally consistent than global SAE. In the main cohort, AKI harm increase was approximately 0.6 percentage points in CKM stage 2, 2.2–2.9 percentage points in CKM stage 3, and 1.6–1.7 percentage points in CKM stage 4 across tertile-median TyG values. Hypotension harm increase ranged from approximately 0.2–0.6 percentage points in CKM stage 2, 0.7–0.8 percentage points in CKM stage 3, and 1.1–2.4 percentage points in CKM stage 4. Safety event counts and Cox-standardized harm-risk differences are provided in Supplementary Table 7.

In exploratory SAE-centered net-benefit analyses, safety-adjusted benefit appeared most preserved among participants with CKM stage 4 and high TyG. For all-cause death in CKM stage 4 with high TyG, ARR was 3.4 percentage points and SAE harm increase was 0.2 percentage points; simplified net benefit remained positive using harm weights of 0.5 and 1.0. In contrast, lower-risk CKM stage 2 strata had small mortality ARR estimates and were more readily offset by harm assumptions. These results were treated as exploratory because global SAE is heterogeneous and the harm weights were sensitivity parameters rather than validated utilities. Exploratory SAE-adjusted simplified net-benefit estimates for all-cause death are summarized in Table 4.

**TABLE 4:** Exploratory SAE-adjusted simplified net-benefit estimates for all-cause death in the main analytic cohort.

CKM stage	TyG tertile	TyG median	Death ARR (95% CI), pp	SAE risk increase (95% CI), pp	Harm weight	Simplified net benefit, pp
CKM stage 2	T1 (low TyG)	8.08	0.0 (−0.9, 1.0)	0.7 (−2.8, 4.3)	0.5	−0.3
CKM stage 2	T2 (middle TyG)	8.56	0.2 (−0.4, 0.8)	1.6 (−0.9, 4.2)	0.5	−0.6
CKM stage 2	T3 (high TyG)	9.11	0.3 (−0.5, 1.2)	2.6 (−1.1, 6.0)	0.5	−1
CKM stage 3	T1 (low TyG)	8.08	−1.3 (−3.1, 0.9)	−1.2 (−6.6, 4.7)	0.5	−0.7
CKM stage 3	T2 (middle TyG)	8.56	−0.2 (−1.5, 1.4)	−0.3 (−5.1, 3.6)	0.5	0
CKM stage 3	T3 (high TyG)	9.11	1.2 (−0.9, 3.4)	0.7 (−5.6, 6.3)	0.5	0.9
CKM stage 4	T1 (low TyG)	8.08	0.7 (−1.1, 2.6)	1.3 (−3.4, 6.1)	0.5	0.1
CKM stage 4	T2 (middle TyG)	8.56	1.9 (0.5, 3.6)	0.8 (−2.5, 4.6)	0.5	1.5
CKM stage 4	T3 (high TyG)	9.11	3.4 (1.3, 5.6)	0.2 (−4.8, 5.6)	0.5	3.2
CKM stage 2	T1 (low TyG)	8.08	0.0 (−0.9, 1.0)	0.7 (−2.8, 4.3)	1	−0.7
CKM stage 2	T2 (middle TyG)	8.56	0.2 (−0.4, 0.8)	1.6 (−0.9, 4.2)	1	−1.4
CKM stage 2	T3 (high TyG)	9.11	0.3 (−0.5, 1.2)	2.6 (−1.1, 6.0)	1	−2.3
CKM stage 3	T1 (low TyG)	8.08	−1.3 (−3.1, 0.9)	−1.2 (−6.6, 4.7)	1	−0.1
CKM stage 3	T2 (middle TyG)	8.56	−0.2 (−1.5, 1.4)	−0.3 (−5.1, 3.6)	1	0.2
CKM stage 3	T3 (high TyG)	9.11	1.2 (−0.9, 3.4)	0.7 (−5.6, 6.3)	1	0.5
CKM stage 4	T1 (low TyG)	8.08	0.7 (−1.1, 2.6)	1.3 (−3.4, 6.1)	1	−0.6
CKM stage 4	T2 (middle TyG)	8.56	1.9 (0.5, 3.6)	0.8 (−2.5, 4.6)	1	1.1
CKM stage 4	T3 (high TyG)	9.11	3.4 (1.3, 5.6)	0.2 (−4.8, 5.6)	1	3.1

Full exploratory safety-adjusted net-benefit analyses, including fasting-only and AKI/hypotension supportive analyses, are provided in Supplementary Table 8.

## 4 Discussion

In this *post hoc* analysis of SPRINT, intensive BP treatment was associated with lower risk of both the primary composite outcome and all-cause death overall. When absolute benefit was examined across CKM stage and TyG strata, the clearest exploratory pattern was observed for all-cause death, particularly among participants with CKM stage 4 and high TyG. In contrast, the primary composite outcome showed less consistent patterns across CKM stage and TyG, although overall intensive BP treatment remained beneficial. Formal interaction testing did not support statistically significant CKM-specific three-way intensive-treatment effect modification; therefore, the findings should be interpreted as exploratory absolute-risk stratification rather than as evidence of a clinically actionable responder subgroup.

These findings highlight the distinction between overall treatment efficacy, interaction-based treatment-effect heterogeneity, and absolute benefit. Intensive BP treatment reduced the primary composite outcome and all-cause death in the adjusted models, consistent with the main SPRINT results (1, 2). However, interaction tests addressed a different question: whether the relative benefit of intensive versus standard BP treatment differed by CKM stage, TyG, or their combination. Evidence for such modification was limited. The nominal intensive BP treatment × TyG interaction for all-cause death suggests a possible association between TyG and variation in absolute mortality benefit, but the absence of a significant CKM stage × TyG × intensive-treatment interaction argues against overinterpreting these findings as CKM-specific treatment-response evidence.

The absolute-risk framework remains clinically informative because the same relative treatment effect can translate into different absolute benefits across baseline-risk strata. In this analysis, TyG was evaluated as an exploratory metabolic marker rather than as a comprehensive measure of CKM metabolic health. Participants with CKM stage 4 and high TyG had the largest estimated all-cause death ARR. This pattern may help describe where greater absolute benefit was observed in SPRINT, but it requires external validation before being used for clinical decision-making.

Safety findings should also be interpreted cautiously. Intensive BP treatment was associated with higher AKI and hypotension harm estimates, whereas global SAE was a broader and less treatment-specific endpoint. Exploratory safety-adjusted analyses suggested that SAE-adjusted benefit appeared most preserved among participants with CKM stage 4 and high TyG, while lower-risk CKM stage 2 strata were more readily offset by harm assumptions. These results provide context for benefit-harm trade-offs but should not be viewed as a formal net clinical benefit model because the harm weights were sensitivity parameters rather than validated patient-centered utilities.

This study has several limitations. It is a *post hoc*, hypothesis-generating analysis of a randomized trial. SPRINT excluded participants with diabetes mellitus and prior stroke, which limits generalizability to the broader CKM population. CKM stage was not prospectively assigned in SPRINT; we implemented the AHA CKM framework using baseline trial variables, as described in Methods section “2.2 CKM staging algorithm.” Importantly, 1,282 of 9,323 TyG-calculable participants remained unclassified because PREVENT-based risk staging could not be performed when required inputs were missing or outside the PREVENT/preventR calculator-supported domain. Excluding these participants avoided forced CKM stage 2 or 3 assignment but may have introduced selection bias and may limit the generalizability of estimates by CKM stage to the full TyG-calculable SPRINT population. The primary models used a parsimonious adjustment set to preserve stability and consistency across efficacy and safety analyses. Expanded-adjustment complete-case sensitivity analyses produced directionally consistent main intensive BP treatment HRs, but CKM/TyG-specific ARR estimates were attenuated and the expanded models had reduced sample size because of additional covariate missingness. Finally, multiple subgroup and interaction analyses increase the possibility of chance findings, and the exploratory safety-adjusted net-benefit analysis requires validation in independent cohorts.

## 5 Conclusion

In this *post hoc* SPRINT analysis, intensive BP treatment reduced overall risk of the primary composite outcome and all-cause death. Exploratory Cox-standardized ARR estimates suggested the greatest absolute mortality benefit among participants with CKM stage 4 and high TyG. Because analyses requiring CKM stage assignment excluded unclassified participants and CKM-specific three-way interaction tests were not statistically significant, these findings should be interpreted as hypothesis-generating absolute-risk stratification rather than as a clinically actionable treatment-response rule.

## Data Availability

Publicly available datasets in the NHLBI BioLINCC (Biologic Specimen and Data Repository Information Coordinating Center) were analyzed in the study: Systolic Blood Pressure Intervention Trial (SPRINT), accession no. HLB02021925a (https://biolincc.nhlbi.nih.gov/studies/sprint/).
