# Supplementary material for: Triglyceride-glucose index, CKM stage, and absolute benefit of intensive blood pressure control: a post hoc analysis of SPRINT
Source: Front Nutr. 2026 Jul 16;13:1859944. doi: 10.3389/fnut.2026.1859944 (PMC13421407; doi:10.3389/fnut.2026.1859944)
Supplement: Supplementary file 1 [file Supplementary_file_1.docx]

**Supplementary Tables and Figures**

*Triglyceride-Glucose Index, CKM Stage, and Absolute Benefit of Intensive Blood Pressure Control: A Post Hoc Analysis of SPRINT*

***Supplementary Table S1. Cohort flow and unclassified-participant reasons.***

**Panel A. Cohort flow.**

| **Cohort-flow step** | **N** | **Note** |
| --- | --- | --- |
| SPRINT randomized participants in Step 3R dataset | 9361 | Total cohort after Step 3R join |
| TyG calculable | 9323 | Participants with calculable TyG |
| TyG missing | 38 | Participants without calculable TyG |
| TyG calculable and classified as CKM 2/3/4 | 8041 | **TyG calculable and classified as CKM stage 2/3/4** Participants assigned CKM stage 2, 3, or 4 by the CKM staging algorithm |
| TyG calculable but unclassified by CKM algorithm | 1282 | **TyG calculable but unclassified by CKM algorithm** Participants who could not be assigned CKM stage 2 or 3 because PREVENT-based risk staging could not be performed |
| TyG calculable but excluded from primary complete-case analytic cohort | 1287 | Includes unclassified CKM plus any missing core model covariates |
| Primary complete-case analytic cohort | 8036 | Main analysis dataset used for adjusted Cox and ARR models |
| Primary complete-case analytic cohort with survival times | 8036 | Check that event time variables are complete |
| Fasting-only sensitivity cohort | 7526 | Sensitivity cohort with fasting status requirement |

**Panel B. Reasons for unclassified CKM status among TyG-calculable participants.**

| **Reason** | **Classification basis** | **N** |
| --- | --- | --- |
| No valid PREVENT output because of out-of-domain non-age PREVENT inputs | PREVENT-based risk staging unavailable | 648 |
| Age outside the 30–79-year PREVENT domain | PREVENT-based risk staging unavailable | 583 |
| Missing BMI | PREVENT-based risk staging unavailable | 42 |
| Missing eGFR | PREVENT-based risk staging unavailable | 9 |

*TyG = triglyceride-glucose index; CKM = cardiovascular-kidney-metabolic; eGFR = estimated glomerular filtration rate. Unclassified participants were not forced into CKM stage 2 or 3 when PREVENT-based risk staging could not be performed. Among the 648 age-eligible participants with no valid PREVENT output, required PREVENT inputs were complete, but at least one non-age input was outside the PREVENT/preventR calculator-supported domain. The most common out-of-domain input was BMI outside 18.5–39.9, followed by total cholesterol, systolic blood pressure, HDL cholesterol, or eGFR outside the supported ranges.*

***Supplementary Table S2. CKM staging algorithm and PREVENT implementation.***

| **Algorithm component** | **Operational rule** | **Details** |
| --- | --- | --- |
| CKM stage 4 | Assigned first using baseline indicators of clinical cardiovascular disease. | Indicators included myocardial infarction/heart attack, acute coronary syndrome, coronary revascularization, carotid disease, peripheral arterial revascularization, atrial fibrillation, angina, congestive heart failure, stroke, and peripheral vascular disease. Participants meeting any CKM4 criterion were classified as CKM4 regardless of PREVENT risk availability. |
| CKM stage 3: non-PREVENT criteria | Assigned among participants not classified as CKM4. | Criteria included very-high-risk CKD according to KDIGO risk classification or a positive cardiovascular disease/subclinical cardiovascular disease history indicator. |
| CKM stage 3: PREVENT risk criterion | Assigned among participants not classified as CKM4 when valid PREVENT risk was available and exceeded the risk threshold. | High predicted risk was defined as PREVENT 10-year total cardiovascular disease risk >=20%. |
| PREVENT implementation | Base PREVENT model implemented in preventR. | Risk-based CKM3 classification required valid inputs within the PREVENT/preventR calculator-supported domain. Required inputs included sex, age, total cholesterol, HDL cholesterol, systolic BP, BMI, eGFR, diabetes status, current smoking status, antihypertensive medication use, and lipid-lowering therapy use. |
| Optional PREVENT inputs | Not included in the primary PREVENT model. | Optional predictors not used in the primary implementation included UACR, HbA1c, and social deprivation index. |
| CKM stage 2 | Assigned after CKM4 and CKM3 rules were evaluated. | Participants not classified as CKM4 or CKM3 and with valid PREVENT risk <20% were classified as CKM stage 2. |
| Unclassified handling | Not forced into CKM stage 2 or 3. | Participants not meeting CKM4 or non-PREVENT CKM3 criteria remained unclassified when PREVENT-based risk staging could not be performed because required inputs were missing or outside the PREVENT/preventR calculator-supported domain. |

*PREVENT risk was used only for the risk-based CKM stage 3 component among participants not classified as CKM stage 4. Optional PREVENT predictors were not used in the primary PREVENT model. This table describes the implementation of the AHA CKM framework using baseline SPRINT variables and should not be interpreted as a new CKM staging framework.*

***Supplementary Table S3. Sex coding audit.***

| **Cohort** | **N** | **Female, n** | **Female, %** | **Male, n** | **Male, %** | **Missing female indicator** | **Female values not 0/1** | **Female + male = N** | **Original sex variable available** | **Sex coding mismatches, n** |
| --- | --- | --- | --- | --- | --- | --- | --- | --- | --- | --- |
| main | 8036 | 2727 | 33.9% | 5309 | 66.1% | 0 | 0 | True | True | 0 |
| fasting_only | 7526 | 2544 | 33.8% | 4982 | 66.2% | 0 | 0 | True | True | 0 |
| all_step3r_program_ckm | 9361 | 3332 | 35.6% | 6029 | 64.4% | 0 | 0 | True | True | 0 |

*Female was coded as 1 and male as 0 in the analysis dataset. The final main analytic cohort included 2,727 female participants (33.9%).*

***Supplementary Table S4. Extended baseline characteristics by CKM stage.***

| **Characteristic** | **Overall (n=8036)** | **CKM2 (n=3718)** | **CKM3 (n=1740)** | **CKM4 (n=2578)** | **P value** |
| --- | --- | --- | --- | --- | --- |
| Age, years | 67.1 ± 8.8 | 62.3 ± 6.1 | 72.5 ± 7.2 | 70.5 ± 9.4 | <0.001 |
| TyG index | 8.60 ± 0.54 | 8.62 ± 0.54 | 8.58 ± 0.53 | 8.57 ± 0.53 | <0.001 |
| Baseline systolic BP, mmHg | 139.1 ± 15.0 | 138.1 ± 14.1 | 141.8 ± 15.1 | 138.8 ± 15.9 | <0.001 |
| Baseline diastolic BP, mmHg | 78.2 ± 11.7 | 81.4 ± 10.4 | 76.0 ± 11.7 | 75.0 ± 12.1 | <0.001 |
| eGFR, mL/min/1.73 m² | 71.6 ± 20.4 | 78.6 ± 17.8 | 62.0 ± 20.3 | 68.0 ± 20.6 | <0.001 |
| Urinary albumin-to-creatinine ratio, mg/g | 9.2 (5.5, 21.0) | 7.6 (5.0, 14.0) | 12.2 (6.6, 37.1) | 11.4 (6.1, 28.9) | <0.001 |
| Triglycerides, mg/dL | 108.0 (78.0, 152.0) | 110.0 (79.0, 157.0) | 106.0 (76.0, 147.0) | 105.0 (76.0, 146.0) | <0.001 |
| Glucose, mg/dL | 98.9 ± 13.5 | 98.6 ± 13.5 | 98.5 ± 13.0 | 99.5 ± 14.0 | 0.002 |
| BMI, kg/m² | 30.0 ± 5.0 | 29.8 ± 4.5 | 29.8 ± 5.3 | 30.5 ± 5.9 | 0.012 |
| Number of baseline BP medications | 1.8 ± 1.0 | 1.6 ± 1.0 | 1.8 ± 1.0 | 2.1 ± 1.0 | <0.001 |
| Female | 2727 (33.9%) | 1400 (37.7%) | 612 (35.2%) | 715 (27.7%) | <0.001 |
| Randomized to intensive BP treatment | 4027 (50.1%) | 1870 (50.3%) | 842 (48.4%) | 1315 (51.0%) | 0.230 |
| White race | 5232 (65.1%) | 2133 (57.4%) | 1193 (68.6%) | 1906 (73.9%) | <0.001 |
| Black race | 2533 (31.5%) | 1460 (39.3%) | 479 (27.5%) | 594 (23.0%) | <0.001 |
| Asian race | 72 (0.9%) | 22 (0.6%) | 27 (1.6%) | 23 (0.9%) | 0.002 |
| Other race | 198 (2.5%) | 97 (2.6%) | 44 (2.5%) | 57 (2.2%) | 0.594 |
| Hispanic ethnicity | 862 (10.7%) | 511 (13.7%) | 158 (9.1%) | 193 (7.5%) | <0.001 |
| Current smoker | 1175 (14.6%) | 652 (17.5%) | 185 (10.6%) | 338 (13.1%) | <0.001 |
| Aspirin use | 4156 (51.7%) | 1444 (38.8%) | 870 (50.0%) | 1842 (71.5%) | <0.001 |

*Values are mean ± SD, median (interquartile range), or n (%). P values compare CKM stages 2–4. Aspirin use is provided descriptively and was not included in the primary adjusted models.*

***Supplementary Table S5. Treatment effects, formal interaction tests, and proportional-hazards diagnostics.***

**Panel A. Main adjusted treatment effects in the main and fasting-only cohorts.**

| **Cohort** | **Outcome** | **Model** | **Term** | **HR (95% CI)** | **P value** |
| --- | --- | --- | --- | --- | --- |
| Fasting-only sensitivity cohort | All-cause death | M1_main_adj | Intensive BP treatment vs standard BP treatment | 0.80 (0.65, 0.97) | 0.026 |
| Fasting-only sensitivity cohort | Primary composite outcome | M1_main_adj | Intensive BP treatment vs standard BP treatment | 0.75 (0.63, 0.88) | <0.001 |
| Main analytic cohort | All-cause death | M1_main_adj | Intensive BP treatment vs standard BP treatment | 0.81 (0.67, 0.98) | 0.031 |
| Main analytic cohort | Primary composite outcome | M1_main_adj | Intensive BP treatment vs standard BP treatment | 0.73 (0.63, 0.86) | <0.001 |

**Panel B. Formal interaction tests in the main and fasting-only cohorts.**

| **Cohort** | **Outcome** | **Interaction test** | **Chi-square** | **df** | **P value** | **Interpretation** |
| --- | --- | --- | --- | --- | --- | --- |
| Fasting-only sensitivity cohort | All-cause death | Intensive BP treatment × CKM stage | 2.02 | 2 | 0.365 | No nominal evidence of interaction |
| Fasting-only sensitivity cohort | All-cause death | Intensive BP treatment × TyG | 4.4 | 1 | 0.036 | Nominal evidence of interaction |
| Fasting-only sensitivity cohort | All-cause death | Intensive BP treatment × CKM stage × TyG | 0.67 | 2 | 0.717 | No nominal evidence of interaction |
| Fasting-only sensitivity cohort | All-cause death | Any intensive-treatment effect modification by CKM stage or TyG | 7.31 | 5 | 0.199 | No nominal evidence of interaction |
| Fasting-only sensitivity cohort | Primary composite outcome | Intensive BP treatment × CKM stage | 1.23 | 2 | 0.542 | No nominal evidence of interaction |
| Fasting-only sensitivity cohort | Primary composite outcome | Intensive BP treatment × TyG | 0.27 | 1 | 0.603 | No nominal evidence of interaction |
| Fasting-only sensitivity cohort | Primary composite outcome | Intensive BP treatment × CKM stage × TyG | 0.9 | 2 | 0.637 | No nominal evidence of interaction |
| Fasting-only sensitivity cohort | Primary composite outcome | Any intensive-treatment effect modification by CKM stage or TyG | 2.38 | 5 | 0.795 | No nominal evidence of interaction |
| Main analytic cohort | All-cause death | Intensive BP treatment × CKM stage | 2.94 | 2 | 0.23 | No nominal evidence of interaction |
| Main analytic cohort | All-cause death | Intensive BP treatment × TyG | 5.04 | 1 | 0.025 | Nominal evidence of interaction |
| Main analytic cohort | All-cause death | Intensive BP treatment × CKM stage × TyG | 0.51 | 2 | 0.775 | No nominal evidence of interaction |
| Main analytic cohort | All-cause death | Any intensive-treatment effect modification by CKM stage or TyG | 8.84 | 5 | 0.116 | No nominal evidence of interaction |
| Main analytic cohort | Primary composite outcome | Intensive BP treatment × CKM stage | 1.49 | 2 | 0.475 | No nominal evidence of interaction |
| Main analytic cohort | Primary composite outcome | Intensive BP treatment × TyG | 0.47 | 1 | 0.491 | No nominal evidence of interaction |
| Main analytic cohort | Primary composite outcome | Intensive BP treatment × CKM stage × TyG | 2.57 | 2 | 0.277 | No nominal evidence of interaction |
| Main analytic cohort | Primary composite outcome | Any intensive-treatment effect modification by CKM stage or TyG | 4.48 | 5 | 0.482 | No nominal evidence of interaction |

*Intensive BP treatment denotes randomized assignment to intensive systolic blood pressure control versus standard control. CKM stage was modeled as a three-level categorical variable with CKM stage 2 as the reference; CKM interaction tests are global likelihood-ratio tests.*

**Panel C. Schoenfeld residual proportional-hazards diagnostics.**

| **Cohort** | **Outcome** | **Model** | **PH diagnostic** | **Term** | **Chi-square** | **df** | **P value** | **Interpretation** |
| --- | --- | --- | --- | --- | --- | --- | --- | --- |
| Fasting-only sensitivity cohort | All-cause death | Full CKM × TyG interaction model | Term-specific Schoenfeld residual test | CKM stage | 7.477 | 2 | 0.0238 | Potential PH departure |
| Fasting-only sensitivity cohort | All-cause death | Full CKM × TyG interaction model | Term-specific Schoenfeld residual test | Intensive BP treatment | 0.571 | 1 | 0.45 | No nominal PH departure |
| Fasting-only sensitivity cohort | All-cause death | Full CKM × TyG interaction model | Term-specific Schoenfeld residual test | Intensive BP treatment × CKM stage | 5.672 | 2 | 0.0587 | No nominal PH departure |
| Fasting-only sensitivity cohort | All-cause death | Full CKM × TyG interaction model | Term-specific Schoenfeld residual test | Intensive BP treatment × CKM stage × TyG | 5.936 | 2 | 0.0514 | No nominal PH departure |
| Fasting-only sensitivity cohort | All-cause death | Full CKM × TyG interaction model | Term-specific Schoenfeld residual test | Intensive BP treatment × TyG | 0.597 | 1 | 0.4399 | No nominal PH departure |
| Fasting-only sensitivity cohort | All-cause death | Full CKM × TyG interaction model | Term-specific Schoenfeld residual test | TyG | 4.643 | 1 | 0.0312 | Potential PH departure |
| Fasting-only sensitivity cohort | All-cause death | Full CKM × TyG interaction model | Global Schoenfeld residual test | Global test | 21.853 | 15 | 0.1117 | No nominal global PH departure |
| Fasting-only sensitivity cohort | All-cause death | Main adjusted model | Term-specific Schoenfeld residual test | CKM stage | 7.429 | 2 | 0.0244 | Potential PH departure |
| Fasting-only sensitivity cohort | All-cause death | Main adjusted model | Term-specific Schoenfeld residual test | Intensive BP treatment | 0.588 | 1 | 0.4433 | No nominal PH departure |
| Fasting-only sensitivity cohort | All-cause death | Main adjusted model | Term-specific Schoenfeld residual test | TyG | 4.744 | 1 | 0.0294 | Potential PH departure |
| Fasting-only sensitivity cohort | All-cause death | Main adjusted model | Global Schoenfeld residual test | Global test | 15.41 | 8 | 0.0516 | No nominal global PH departure |
| Fasting-only sensitivity cohort | Primary composite outcome | Full CKM × TyG interaction model | Term-specific Schoenfeld residual test | CKM stage | 0.232 | 2 | 0.8906 | No nominal PH departure |
| Fasting-only sensitivity cohort | Primary composite outcome | Full CKM × TyG interaction model | Term-specific Schoenfeld residual test | Intensive BP treatment | 0.776 | 1 | 0.3785 | No nominal PH departure |
| Fasting-only sensitivity cohort | Primary composite outcome | Full CKM × TyG interaction model | Term-specific Schoenfeld residual test | Intensive BP treatment × CKM stage | 4.552 | 2 | 0.1027 | No nominal PH departure |
| Fasting-only sensitivity cohort | Primary composite outcome | Full CKM × TyG interaction model | Term-specific Schoenfeld residual test | Intensive BP treatment × CKM stage × TyG | 4.515 | 2 | 0.1046 | No nominal PH departure |
| Fasting-only sensitivity cohort | Primary composite outcome | Full CKM × TyG interaction model | Term-specific Schoenfeld residual test | Intensive BP treatment × TyG | 0.723 | 1 | 0.395 | No nominal PH departure |
| Fasting-only sensitivity cohort | Primary composite outcome | Full CKM × TyG interaction model | Term-specific Schoenfeld residual test | TyG | 0 | 1 | 0.9981 | No nominal PH departure |
| Fasting-only sensitivity cohort | Primary composite outcome | Full CKM × TyG interaction model | Global Schoenfeld residual test | Global test | 15.075 | 15 | 0.4461 | No nominal global PH departure |
| Fasting-only sensitivity cohort | Primary composite outcome | Main adjusted model | Term-specific Schoenfeld residual test | CKM stage | 0.228 | 2 | 0.8924 | No nominal PH departure |
| Fasting-only sensitivity cohort | Primary composite outcome | Main adjusted model | Term-specific Schoenfeld residual test | Intensive BP treatment | 0.831 | 1 | 0.3619 | No nominal PH departure |
| Fasting-only sensitivity cohort | Primary composite outcome | Main adjusted model | Term-specific Schoenfeld residual test | TyG | 0.001 | 1 | 0.9789 | No nominal PH departure |
| Fasting-only sensitivity cohort | Primary composite outcome | Main adjusted model | Global Schoenfeld residual test | Global test | 4.654 | 8 | 0.7938 | No nominal global PH departure |
| Main analytic cohort | All-cause death | Full CKM × TyG interaction model | Term-specific Schoenfeld residual test | CKM stage | 5.295 | 2 | 0.0708 | No nominal PH departure |
| Main analytic cohort | All-cause death | Full CKM × TyG interaction model | Term-specific Schoenfeld residual test | Intensive BP treatment | 1.091 | 1 | 0.2963 | No nominal PH departure |
| Main analytic cohort | All-cause death | Full CKM × TyG interaction model | Term-specific Schoenfeld residual test | Intensive BP treatment × CKM stage | 4.27 | 2 | 0.1183 | No nominal PH departure |
| Main analytic cohort | All-cause death | Full CKM × TyG interaction model | Term-specific Schoenfeld residual test | Intensive BP treatment × CKM stage × TyG | 4.379 | 2 | 0.112 | No nominal PH departure |
| Main analytic cohort | All-cause death | Full CKM × TyG interaction model | Term-specific Schoenfeld residual test | Intensive BP treatment × TyG | 1.16 | 1 | 0.2815 | No nominal PH departure |
| Main analytic cohort | All-cause death | Full CKM × TyG interaction model | Term-specific Schoenfeld residual test | TyG | 5.307 | 1 | 0.0212 | Potential PH departure |
| Main analytic cohort | All-cause death | Full CKM × TyG interaction model | Global Schoenfeld residual test | Global test | 22.217 | 15 | 0.1022 | No nominal global PH departure |
| Main analytic cohort | All-cause death | Main adjusted model | Term-specific Schoenfeld residual test | CKM stage | 5.279 | 2 | 0.0714 | No nominal PH departure |
| Main analytic cohort | All-cause death | Main adjusted model | Term-specific Schoenfeld residual test | Intensive BP treatment | 1.13 | 1 | 0.2878 | No nominal PH departure |
| Main analytic cohort | All-cause death | Main adjusted model | Term-specific Schoenfeld residual test | TyG | 5.512 | 1 | 0.0189 | Potential PH departure |
| Main analytic cohort | All-cause death | Main adjusted model | Global Schoenfeld residual test | Global test | 15.574 | 8 | 0.0489 | Potential global PH departure |
| Main analytic cohort | Primary composite outcome | Full CKM × TyG interaction model | Term-specific Schoenfeld residual test | CKM stage | 0.437 | 2 | 0.8039 | No nominal PH departure |
| Main analytic cohort | Primary composite outcome | Full CKM × TyG interaction model | Term-specific Schoenfeld residual test | Intensive BP treatment | 1.075 | 1 | 0.2997 | No nominal PH departure |
| Main analytic cohort | Primary composite outcome | Full CKM × TyG interaction model | Term-specific Schoenfeld residual test | Intensive BP treatment × CKM stage | 4.668 | 2 | 0.0969 | No nominal PH departure |
| Main analytic cohort | Primary composite outcome | Full CKM × TyG interaction model | Term-specific Schoenfeld residual test | Intensive BP treatment × CKM stage × TyG | 4.493 | 2 | 0.1058 | No nominal PH departure |
| Main analytic cohort | Primary composite outcome | Full CKM × TyG interaction model | Term-specific Schoenfeld residual test | Intensive BP treatment × TyG | 0.98 | 1 | 0.3223 | No nominal PH departure |
| Main analytic cohort | Primary composite outcome | Full CKM × TyG interaction model | Term-specific Schoenfeld residual test | TyG | 0.001 | 1 | 0.9693 | No nominal PH departure |
| Main analytic cohort | Primary composite outcome | Full CKM × TyG interaction model | Global Schoenfeld residual test | Global test | 16.074 | 15 | 0.3771 | No nominal global PH departure |
| Main analytic cohort | Primary composite outcome | Main adjusted model | Term-specific Schoenfeld residual test | CKM stage | 0.448 | 2 | 0.7993 | No nominal PH departure |
| Main analytic cohort | Primary composite outcome | Main adjusted model | Term-specific Schoenfeld residual test | Intensive BP treatment | 1.141 | 1 | 0.2854 | No nominal PH departure |
| Main analytic cohort | Primary composite outcome | Main adjusted model | Term-specific Schoenfeld residual test | TyG | 0.005 | 1 | 0.9431 | No nominal PH departure |
| Main analytic cohort | Primary composite outcome | Main adjusted model | Global Schoenfeld residual test | Global test | 4.6 | 8 | 0.7993 | No nominal global PH departure |

*Proportional-hazards diagnostics were based on Schoenfeld residual tests. P<0.05 was considered nominal evidence of proportional-hazards departure. Intensive BP treatment denotes randomized assignment to intensive systolic blood pressure control versus standard control.*

***Supplementary Table S6. Cox-standardized 3-year ARR estimates in main and fasting-only cohorts.***

**Panel A. Main analytic cohort.**

| **Outcome** | **CKM stage** | **TyG tertile** | **TyG median** | **Tertile range** | **3-year risk, standard/intensive (%)** | **ARR, percentage points (95% CI)** | **Bootstrap successful** |
| --- | --- | --- | --- | --- | --- | --- | --- |
| All-cause death | CKM stage 2 | T1 (low TyG) | 8.08 | 6.74–8.34 | 1.3 / 1.3 | 0.0 (-0.9, 1.0) | 300 |
| All-cause death | CKM stage 2 | T2 (middle TyG) | 8.56 | 8.34–8.80 | 1.4 / 1.2 | 0.2 (-0.4, 0.8) | 300 |
| All-cause death | CKM stage 2 | T3 (high TyG) | 9.11 | 8.80–11.32 | 1.4 / 1.2 | 0.3 (-0.5, 1.2) | 300 |
| All-cause death | CKM stage 3 | T1 (low TyG) | 8.08 | 6.74–8.34 | 3.5 / 4.8 | -1.3 (-3.1, 0.9) | 300 |
| All-cause death | CKM stage 3 | T2 (middle TyG) | 8.56 | 8.34–8.80 | 4.3 / 4.5 | -0.2 (-1.5, 1.4) | 300 |
| All-cause death | CKM stage 3 | T3 (high TyG) | 9.11 | 8.80–11.32 | 5.3 / 4.1 | 1.2 (-0.9, 3.4) | 300 |
| All-cause death | CKM stage 4 | T1 (low TyG) | 8.08 | 6.74–8.34 | 5.7 / 5.0 | 0.7 (-1.1, 2.6) | 300 |
| All-cause death | CKM stage 4 | T2 (middle TyG) | 8.56 | 8.34–8.80 | 6.5 / 4.5 | 1.9 (0.5, 3.6) | 300 |
| All-cause death | CKM stage 4 | T3 (high TyG) | 9.11 | 8.80–11.32 | 7.4 / 4.1 | 3.4 (1.3, 5.6) | 300 |
| Primary composite outcome | CKM stage 2 | T1 (low TyG) | 8.08 | 6.74–8.34 | 2.7 / 1.7 | 1.1 (-0.0, 2.2) | 300 |
| Primary composite outcome | CKM stage 2 | T2 (middle TyG) | 8.56 | 8.34–8.80 | 3.1 / 1.9 | 1.2 (0.4, 2.1) | 300 |
| Primary composite outcome | CKM stage 2 | T3 (high TyG) | 9.11 | 8.80–11.32 | 3.6 / 2.2 | 1.4 (0.2, 2.7) | 300 |
| Primary composite outcome | CKM stage 3 | T1 (low TyG) | 8.08 | 6.74–8.34 | 4.9 / 5.3 | -0.3 (-2.8, 2.0) | 300 |
| Primary composite outcome | CKM stage 3 | T2 (middle TyG) | 8.56 | 8.34–8.80 | 6.3 / 5.2 | 1.1 (-0.7, 3.1) | 300 |
| Primary composite outcome | CKM stage 3 | T3 (high TyG) | 9.11 | 8.80–11.32 | 8.3 / 5.1 | 3.2 (0.6, 5.9) | 300 |
| Primary composite outcome | CKM stage 4 | T1 (low TyG) | 8.08 | 6.74–8.34 | 10.9 / 8.3 | 2.6 (0.0, 5.3) | 300 |
| Primary composite outcome | CKM stage 4 | T2 (middle TyG) | 8.56 | 8.34–8.80 | 12.6 / 9.8 | 2.8 (0.8, 4.8) | 300 |
| Primary composite outcome | CKM stage 4 | T3 (high TyG) | 9.11 | 8.80–11.32 | 14.7 / 11.7 | 3.0 (-0.4, 6.5) | 300 |

**Panel B. Fasting-only sensitivity cohort.**

| **Outcome** | **CKM stage** | **TyG tertile** | **TyG median** | **Tertile range** | **3-year risk, standard/intensive (%)** | **ARR, percentage points (95% CI)** | **NNT** | **Bootstrap successful** |
| --- | --- | --- | --- | --- | --- | --- | --- | --- |
| All-cause death | CKM stage 2 | T1 (low TyG) | 8.08 | 7.04–8.34 | 1.4 / 1.3 | 0.1 (-0.7, 1.0) | 881.0 | 300 |
| All-cause death | CKM stage 2 | T2 (middle TyG) | 8.56 | 8.34–8.79 | 1.5 / 1.3 | 0.2 (-0.4, 0.9) | 498.0 | 300 |
| All-cause death | CKM stage 2 | T3 (high TyG) | 9.11 | 8.79–11.32 | 1.6 / 1.2 | 0.3 (-0.6, 1.1) | 329.0 | 300 |
| All-cause death | CKM stage 3 | T1 (low TyG) | 8.08 | 7.04–8.34 | 3.7 / 4.7 | -1.0 (-2.9, 0.7) |  | 300 |
| All-cause death | CKM stage 3 | T2 (middle TyG) | 8.56 | 8.34–8.79 | 4.3 / 4.2 | 0.1 (-1.4, 1.7) | 1121.0 | 300 |
| All-cause death | CKM stage 3 | T3 (high TyG) | 9.11 | 8.79–11.32 | 5.1 / 3.7 | 1.4 (-0.9, 3.5) | 70.0 | 300 |
| All-cause death | CKM stage 4 | T1 (low TyG) | 8.08 | 7.04–8.34 | 5.4 / 4.7 | 0.7 (-1.2, 2.7) | 143.0 | 300 |
| All-cause death | CKM stage 4 | T2 (middle TyG) | 8.56 | 8.34–8.79 | 6.1 / 4.2 | 1.8 (0.3, 3.3) | 55.0 | 300 |
| All-cause death | CKM stage 4 | T3 (high TyG) | 9.11 | 8.79–11.32 | 6.9 / 3.7 | 3.1 (0.7, 5.3) | 32.0 | 300 |
| Primary composite outcome | CKM stage 2 | T1 (low TyG) | 8.08 | 7.04–8.34 | 2.8 / 1.7 | 1.1 (-0.0, 2.3) | 89.0 | 300 |
| Primary composite outcome | CKM stage 2 | T2 (middle TyG) | 8.56 | 8.34–8.79 | 3.1 / 1.9 | 1.2 (0.3, 2.0) | 85.0 | 300 |
| Primary composite outcome | CKM stage 2 | T3 (high TyG) | 9.11 | 8.79–11.32 | 3.4 / 2.2 | 1.2 (-0.1, 2.6) | 81.0 | 300 |
| Primary composite outcome | CKM stage 3 | T1 (low TyG) | 8.08 | 7.04–8.34 | 4.9 / 4.7 | 0.2 (-2.2, 2.4) | 409.0 | 300 |
| Primary composite outcome | CKM stage 3 | T2 (middle TyG) | 8.56 | 8.34–8.79 | 6.1 / 4.9 | 1.2 (-0.7, 3.0) | 84.0 | 300 |
| Primary composite outcome | CKM stage 3 | T3 (high TyG) | 9.11 | 8.79–11.32 | 7.8 / 5.2 | 2.6 (-0.1, 5.1) | 39.0 | 300 |
| Primary composite outcome | CKM stage 4 | T1 (low TyG) | 8.08 | 7.04–8.34 | 10.3 / 8.3 | 2.0 (-0.7, 4.7) | 50.0 | 300 |
| Primary composite outcome | CKM stage 4 | T2 (middle TyG) | 8.56 | 8.34–8.79 | 11.9 / 9.5 | 2.4 (0.4, 4.4) | 42.0 | 300 |
| Primary composite outcome | CKM stage 4 | T3 (high TyG) | 9.11 | 8.79–11.32 | 14.1 / 11.1 | 2.9 (-0.2, 6.3) | 34.0 | 300 |

*ARR = absolute risk reduction, defined as standardized risk under standard BP treatment minus standardized risk under intensive BP treatment. Positive values favor intensive BP treatment. NNT display rules are provided in Supplementary Table S9.*

***Supplementary Figure S1. Fasting-only sensitivity analysis: Cox-standardized 3-year ARR for the primary composite outcome across continuous TyG by CKM stage.***


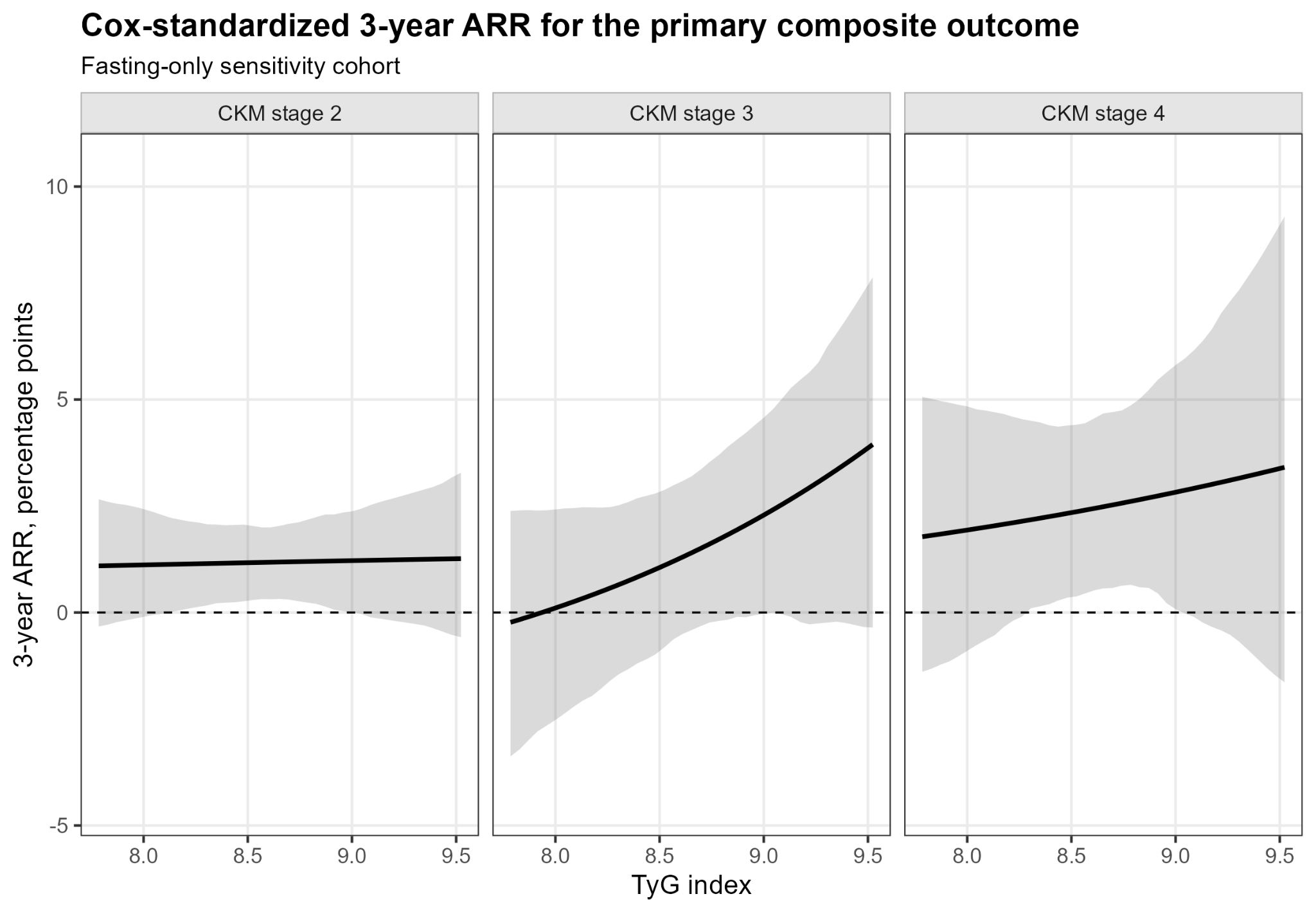


*ARR = absolute risk reduction. Shaded bands denote percentile bootstrap 95% confidence intervals.*

***Supplementary Figure S2. Fasting-only sensitivity analysis: Cox-standardized 3-year ARR for all-cause death across continuous TyG by CKM stage.***


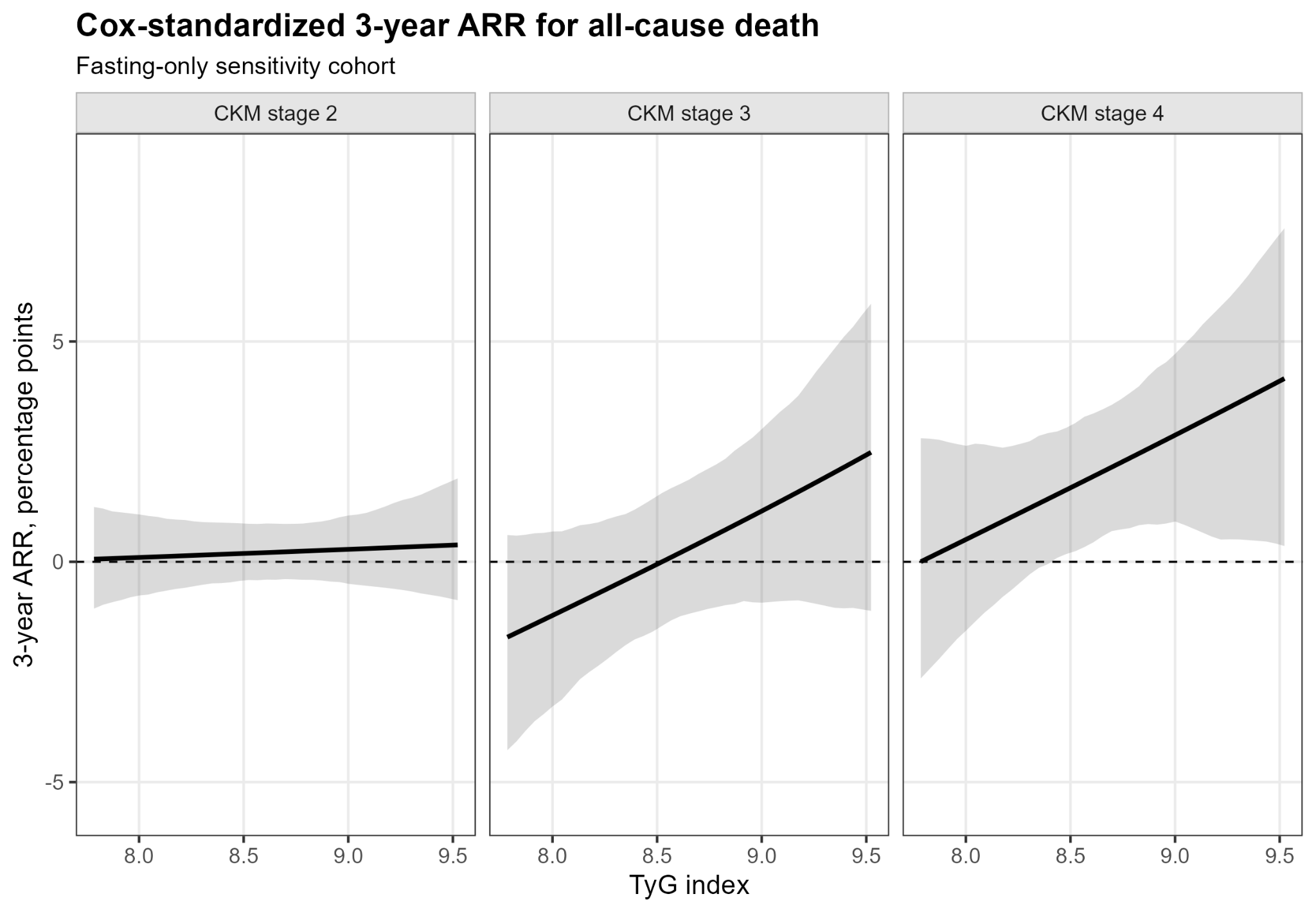


*ARR = absolute risk reduction. Shaded bands denote percentile bootstrap 95% confidence intervals.*

***Supplementary Table S7. Cox-standardized safety harm risk differences in the main analytic cohort.***

| **Harm** | **CKM stage** | **TyG tertile** | **TyG median** | **3-year harm risk, standard/intensive (%)** | **Harm increase, percentage points (95% CI)** | **Bootstrap successful** |
| --- | --- | --- | --- | --- | --- | --- |
| Serious adverse event | CKM stage 2 | T1 (low TyG) | 8.08 | 23.4 / 24.1 | 0.7 (-2.8, 4.3) | 300 |
| Serious adverse event | CKM stage 2 | T2 (middle TyG) | 8.56 | 23.1 / 24.7 | 1.6 (-0.9, 4.2) | 300 |
| Serious adverse event | CKM stage 2 | T3 (high TyG) | 9.11 | 22.7 / 25.4 | 2.6 (-1.1, 6.0) | 300 |
| Serious adverse event | CKM stage 3 | T1 (low TyG) | 8.08 | 36.6 / 35.4 | -1.2 (-6.6, 4.7) | 300 |
| Serious adverse event | CKM stage 3 | T2 (middle TyG) | 8.56 | 37.1 / 36.7 | -0.3 (-5.1, 3.6) | 300 |
| Serious adverse event | CKM stage 3 | T3 (high TyG) | 9.11 | 37.7 / 38.3 | 0.7 (-5.6, 6.3) | 300 |
| Serious adverse event | CKM stage 4 | T1 (low TyG) | 8.08 | 48.0 / 49.3 | 1.3 (-3.4, 6.1) | 300 |
| Serious adverse event | CKM stage 4 | T2 (middle TyG) | 8.56 | 48.6 / 49.4 | 0.8 (-2.5, 4.6) | 300 |
| Serious adverse event | CKM stage 4 | T3 (high TyG) | 9.11 | 49.3 / 49.6 | 0.2 (-4.8, 5.6) | 300 |
| Acute kidney injury SAE | CKM stage 2 | T1 (low TyG) | 8.08 | 1.1 / 1.7 | 0.6 (-0.4, 1.7) | 300 |
| Acute kidney injury SAE | CKM stage 2 | T2 (middle TyG) | 8.56 | 1.2 / 1.8 | 0.6 (-0.1, 1.4) | 300 |
| Acute kidney injury SAE | CKM stage 2 | T3 (high TyG) | 9.11 | 1.3 / 1.9 | 0.6 (-0.3, 1.6) | 300 |
| Acute kidney injury SAE | CKM stage 3 | T1 (low TyG) | 8.08 | 2.3 / 5.3 | 2.9 (0.5, 5.6) | 300 |
| Acute kidney injury SAE | CKM stage 3 | T2 (middle TyG) | 8.56 | 2.8 / 5.4 | 2.6 (1.0, 4.2) | 300 |
| Acute kidney injury SAE | CKM stage 3 | T3 (high TyG) | 9.11 | 3.4 / 5.5 | 2.2 (-0.2, 4.6) | 300 |
| Acute kidney injury SAE | CKM stage 4 | T1 (low TyG) | 8.08 | 4.6 / 6.1 | 1.6 (-0.8, 3.6) | 300 |
| Acute kidney injury SAE | CKM stage 4 | T2 (middle TyG) | 8.56 | 4.0 / 5.6 | 1.7 (0.2, 3.2) | 300 |
| Acute kidney injury SAE | CKM stage 4 | T3 (high TyG) | 9.11 | 3.3 / 5.1 | 1.7 (-0.3, 3.8) | 300 |
| Hypotension SAE | CKM stage 2 | T1 (low TyG) | 8.08 | 0.8 / 1.4 | 0.6 (-0.3, 1.5) | 300 |
| Hypotension SAE | CKM stage 2 | T2 (middle TyG) | 8.56 | 0.9 / 1.3 | 0.4 (-0.2, 1.1) | 300 |
| Hypotension SAE | CKM stage 2 | T3 (high TyG) | 9.11 | 1.0 / 1.3 | 0.2 (-0.6, 1.2) | 300 |
| Hypotension SAE | CKM stage 3 | T1 (low TyG) | 8.08 | 1.8 / 2.6 | 0.8 (-1.3, 2.4) | 300 |
| Hypotension SAE | CKM stage 3 | T2 (middle TyG) | 8.56 | 1.5 / 2.2 | 0.7 (-0.5, 1.8) | 300 |
| Hypotension SAE | CKM stage 3 | T3 (high TyG) | 9.11 | 1.1 / 1.8 | 0.7 (-0.9, 2.0) | 300 |
| Hypotension SAE | CKM stage 4 | T1 (low TyG) | 8.08 | 2.5 / 3.6 | 1.1 (-0.5, 2.8) | 300 |
| Hypotension SAE | CKM stage 4 | T2 (middle TyG) | 8.56 | 2.1 / 3.8 | 1.8 (0.6, 3.0) | 300 |
| Hypotension SAE | CKM stage 4 | T3 (high TyG) | 9.11 | 1.6 / 4.1 | 2.4 (0.7, 4.2) | 300 |

*Harm increase is defined as the predicted 3-year harm risk under intensive BP treatment minus the corresponding risk under standard BP treatment. In the main cohort, global SAE events occurred in 2,992 participants, acute kidney injury SAE events in 272, and hypotension SAE events in 160.*

***Supplementary Table S8. Exploratory safety-adjusted net-benefit analyses.***

**Panel A. SAE-centered simplified net-benefit estimates in the main analytic cohort.**

| **Outcome** | **CKM stage** | **TyG tertile** | **TyG median** | **Benefit ARR, percentage points (95% CI)** | **SAE risk increase, percentage points (95% CI)** | **Harm weight** | **Simplified net benefit, percentage points** |
| --- | --- | --- | --- | --- | --- | --- | --- |
| All-cause death | CKM stage 2 | T1 (low TyG) | 8.08 | 0.0 (-0.9, 1.0) | 0.7 (-2.8, 4.3) | 0.5 | -0.3 |
| All-cause death | CKM stage 2 | T2 (middle TyG) | 8.56 | 0.2 (-0.4, 0.8) | 1.6 (-0.9, 4.2) | 0.5 | -0.6 |
| All-cause death | CKM stage 2 | T3 (high TyG) | 9.11 | 0.3 (-0.5, 1.2) | 2.6 (-1.1, 6.0) | 0.5 | -1.0 |
| All-cause death | CKM stage 3 | T1 (low TyG) | 8.08 | -1.3 (-3.1, 0.9) | -1.2 (-6.6, 4.7) | 0.5 | -0.7 |
| All-cause death | CKM stage 3 | T2 (middle TyG) | 8.56 | -0.2 (-1.5, 1.4) | -0.3 (-5.1, 3.6) | 0.5 | -0.0 |
| All-cause death | CKM stage 3 | T3 (high TyG) | 9.11 | 1.2 (-0.9, 3.4) | 0.7 (-5.6, 6.3) | 0.5 | 0.9 |
| All-cause death | CKM stage 4 | T1 (low TyG) | 8.08 | 0.7 (-1.1, 2.6) | 1.3 (-3.4, 6.1) | 0.5 | 0.1 |
| All-cause death | CKM stage 4 | T2 (middle TyG) | 8.56 | 1.9 (0.5, 3.6) | 0.8 (-2.5, 4.6) | 0.5 | 1.5 |
| All-cause death | CKM stage 4 | T3 (high TyG) | 9.11 | 3.4 (1.3, 5.6) | 0.2 (-4.8, 5.6) | 0.5 | 3.2 |
| All-cause death | CKM stage 2 | T1 (low TyG) | 8.08 | 0.0 (-0.9, 1.0) | 0.7 (-2.8, 4.3) | 1.0 | -0.7 |
| All-cause death | CKM stage 2 | T2 (middle TyG) | 8.56 | 0.2 (-0.4, 0.8) | 1.6 (-0.9, 4.2) | 1.0 | -1.4 |
| All-cause death | CKM stage 2 | T3 (high TyG) | 9.11 | 0.3 (-0.5, 1.2) | 2.6 (-1.1, 6.0) | 1.0 | -2.3 |
| All-cause death | CKM stage 3 | T1 (low TyG) | 8.08 | -1.3 (-3.1, 0.9) | -1.2 (-6.6, 4.7) | 1.0 | -0.1 |
| All-cause death | CKM stage 3 | T2 (middle TyG) | 8.56 | -0.2 (-1.5, 1.4) | -0.3 (-5.1, 3.6) | 1.0 | 0.2 |
| All-cause death | CKM stage 3 | T3 (high TyG) | 9.11 | 1.2 (-0.9, 3.4) | 0.7 (-5.6, 6.3) | 1.0 | 0.5 |
| All-cause death | CKM stage 4 | T1 (low TyG) | 8.08 | 0.7 (-1.1, 2.6) | 1.3 (-3.4, 6.1) | 1.0 | -0.6 |
| All-cause death | CKM stage 4 | T2 (middle TyG) | 8.56 | 1.9 (0.5, 3.6) | 0.8 (-2.5, 4.6) | 1.0 | 1.1 |
| All-cause death | CKM stage 4 | T3 (high TyG) | 9.11 | 3.4 (1.3, 5.6) | 0.2 (-4.8, 5.6) | 1.0 | 3.1 |
| Primary composite outcome | CKM stage 2 | T1 (low TyG) | 8.08 | 1.1 (-0.0, 2.2) | 0.7 (-2.8, 4.3) | 0.5 | 0.7 |
| Primary composite outcome | CKM stage 2 | T2 (middle TyG) | 8.56 | 1.2 (0.4, 2.1) | 1.6 (-0.9, 4.2) | 0.5 | 0.4 |
| Primary composite outcome | CKM stage 2 | T3 (high TyG) | 9.11 | 1.4 (0.2, 2.7) | 2.6 (-1.1, 6.0) | 0.5 | 0.1 |
| Primary composite outcome | CKM stage 3 | T1 (low TyG) | 8.08 | -0.3 (-2.8, 2.0) | -1.2 (-6.6, 4.7) | 0.5 | 0.3 |
| Primary composite outcome | CKM stage 3 | T2 (middle TyG) | 8.56 | 1.1 (-0.7, 3.1) | -0.3 (-5.1, 3.6) | 0.5 | 1.3 |
| Primary composite outcome | CKM stage 3 | T3 (high TyG) | 9.11 | 3.2 (0.6, 5.9) | 0.7 (-5.6, 6.3) | 0.5 | 2.8 |
| Primary composite outcome | CKM stage 4 | T1 (low TyG) | 8.08 | 2.6 (0.0, 5.3) | 1.3 (-3.4, 6.1) | 0.5 | 2.0 |
| Primary composite outcome | CKM stage 4 | T2 (middle TyG) | 8.56 | 2.8 (0.8, 4.8) | 0.8 (-2.5, 4.6) | 0.5 | 2.4 |
| Primary composite outcome | CKM stage 4 | T3 (high TyG) | 9.11 | 3.0 (-0.4, 6.5) | 0.2 (-4.8, 5.6) | 0.5 | 2.9 |
| Primary composite outcome | CKM stage 2 | T1 (low TyG) | 8.08 | 1.1 (-0.0, 2.2) | 0.7 (-2.8, 4.3) | 1.0 | 0.4 |
| Primary composite outcome | CKM stage 2 | T2 (middle TyG) | 8.56 | 1.2 (0.4, 2.1) | 1.6 (-0.9, 4.2) | 1.0 | -0.4 |
| Primary composite outcome | CKM stage 2 | T3 (high TyG) | 9.11 | 1.4 (0.2, 2.7) | 2.6 (-1.1, 6.0) | 1.0 | -1.2 |
| Primary composite outcome | CKM stage 3 | T1 (low TyG) | 8.08 | -0.3 (-2.8, 2.0) | -1.2 (-6.6, 4.7) | 1.0 | 0.8 |
| Primary composite outcome | CKM stage 3 | T2 (middle TyG) | 8.56 | 1.1 (-0.7, 3.1) | -0.3 (-5.1, 3.6) | 1.0 | 1.4 |
| Primary composite outcome | CKM stage 3 | T3 (high TyG) | 9.11 | 3.2 (0.6, 5.9) | 0.7 (-5.6, 6.3) | 1.0 | 2.5 |
| Primary composite outcome | CKM stage 4 | T1 (low TyG) | 8.08 | 2.6 (0.0, 5.3) | 1.3 (-3.4, 6.1) | 1.0 | 1.3 |
| Primary composite outcome | CKM stage 4 | T2 (middle TyG) | 8.56 | 2.8 (0.8, 4.8) | 0.8 (-2.5, 4.6) | 1.0 | 2.0 |
| Primary composite outcome | CKM stage 4 | T3 (high TyG) | 9.11 | 3.0 (-0.4, 6.5) | 0.2 (-4.8, 5.6) | 1.0 | 2.8 |

**Panel B. Supportive net-benefit estimates using acute kidney injury SAE or hypotension SAE as harm components.**

| **Cohort** | **Outcome** | **Harm** | **CKM stage** | **TyG tertile** | **TyG median** | **Benefit ARR, percentage points (95% CI)** | **Harm risk increase, percentage points (95% CI)** | **Harm weight** | **Simplified net benefit, percentage points** |
| --- | --- | --- | --- | --- | --- | --- | --- | --- | --- |
| fasting_only | All-cause death | Acute kidney injury SAE | CKM stage 2 | T1 (low TyG) | 8.08 | 0.1 (-0.7, 1.0) | 0.6 (-0.3, 1.7) | 0.5 | -0.2 |
| fasting_only | All-cause death | Acute kidney injury SAE | CKM stage 2 | T2 (middle TyG) | 8.56 | 0.2 (-0.4, 0.9) | 0.6 (-0.1, 1.3) | 0.5 | -0.1 |
| fasting_only | All-cause death | Acute kidney injury SAE | CKM stage 2 | T3 (high TyG) | 9.11 | 0.3 (-0.6, 1.1) | 0.6 (-0.5, 1.6) | 0.5 | 0.0 |
| fasting_only | All-cause death | Acute kidney injury SAE | CKM stage 3 | T1 (low TyG) | 8.08 | -1.0 (-2.9, 0.7) | 2.6 (0.4, 5.2) | 0.5 | -2.3 |
| fasting_only | All-cause death | Acute kidney injury SAE | CKM stage 3 | T2 (middle TyG) | 8.56 | 0.1 (-1.4, 1.7) | 2.5 (0.8, 4.0) | 0.5 | -1.1 |
| fasting_only | All-cause death | Acute kidney injury SAE | CKM stage 3 | T3 (high TyG) | 9.11 | 1.4 (-0.9, 3.5) | 2.2 (-0.6, 4.9) | 0.5 | 0.3 |
| fasting_only | All-cause death | Acute kidney injury SAE | CKM stage 4 | T1 (low TyG) | 8.08 | 0.7 (-1.2, 2.7) | 1.0 (-1.4, 3.3) | 0.5 | 0.2 |
| fasting_only | All-cause death | Acute kidney injury SAE | CKM stage 4 | T2 (middle TyG) | 8.56 | 1.8 (0.3, 3.3) | 1.5 (-0.1, 3.0) | 0.5 | 1.1 |
| fasting_only | All-cause death | Acute kidney injury SAE | CKM stage 4 | T3 (high TyG) | 9.11 | 3.1 (0.7, 5.3) | 2.0 (-0.1, 4.1) | 0.5 | 2.2 |
| fasting_only | All-cause death | Acute kidney injury SAE | CKM stage 2 | T1 (low TyG) | 8.08 | 0.1 (-0.7, 1.0) | 0.6 (-0.3, 1.7) | 1.0 | -0.5 |
| fasting_only | All-cause death | Acute kidney injury SAE | CKM stage 2 | T2 (middle TyG) | 8.56 | 0.2 (-0.4, 0.9) | 0.6 (-0.1, 1.3) | 1.0 | -0.4 |
| fasting_only | All-cause death | Acute kidney injury SAE | CKM stage 2 | T3 (high TyG) | 9.11 | 0.3 (-0.6, 1.1) | 0.6 (-0.5, 1.6) | 1.0 | -0.3 |
| fasting_only | All-cause death | Acute kidney injury SAE | CKM stage 3 | T1 (low TyG) | 8.08 | -1.0 (-2.9, 0.7) | 2.6 (0.4, 5.2) | 1.0 | -3.6 |
| fasting_only | All-cause death | Acute kidney injury SAE | CKM stage 3 | T2 (middle TyG) | 8.56 | 0.1 (-1.4, 1.7) | 2.5 (0.8, 4.0) | 1.0 | -2.4 |
| fasting_only | All-cause death | Acute kidney injury SAE | CKM stage 3 | T3 (high TyG) | 9.11 | 1.4 (-0.9, 3.5) | 2.2 (-0.6, 4.9) | 1.0 | -0.8 |
| fasting_only | All-cause death | Acute kidney injury SAE | CKM stage 4 | T1 (low TyG) | 8.08 | 0.7 (-1.2, 2.7) | 1.0 (-1.4, 3.3) | 1.0 | -0.3 |
| fasting_only | All-cause death | Acute kidney injury SAE | CKM stage 4 | T2 (middle TyG) | 8.56 | 1.8 (0.3, 3.3) | 1.5 (-0.1, 3.0) | 1.0 | 0.3 |
| fasting_only | All-cause death | Acute kidney injury SAE | CKM stage 4 | T3 (high TyG) | 9.11 | 3.1 (0.7, 5.3) | 2.0 (-0.1, 4.1) | 1.0 | 1.2 |
| fasting_only | All-cause death | Hypotension SAE | CKM stage 2 | T1 (low TyG) | 8.08 | 0.1 (-0.7, 1.0) | 0.6 (-0.3, 1.6) | 0.5 | -0.2 |
| fasting_only | All-cause death | Hypotension SAE | CKM stage 2 | T2 (middle TyG) | 8.56 | 0.2 (-0.4, 0.9) | 0.4 (-0.2, 1.0) | 0.5 | -0.0 |
| fasting_only | All-cause death | Hypotension SAE | CKM stage 2 | T3 (high TyG) | 9.11 | 0.3 (-0.6, 1.1) | 0.3 (-0.5, 1.1) | 0.5 | 0.2 |
| fasting_only | All-cause death | Hypotension SAE | CKM stage 3 | T1 (low TyG) | 8.08 | -1.0 (-2.9, 0.7) | 0.8 (-1.0, 2.5) | 0.5 | -1.4 |
| fasting_only | All-cause death | Hypotension SAE | CKM stage 3 | T2 (middle TyG) | 8.56 | 0.1 (-1.4, 1.7) | 0.8 (-0.5, 1.9) | 0.5 | -0.3 |
| fasting_only | All-cause death | Hypotension SAE | CKM stage 3 | T3 (high TyG) | 9.11 | 1.4 (-0.9, 3.5) | 0.8 (-0.9, 2.3) | 0.5 | 1.0 |
| fasting_only | All-cause death | Hypotension SAE | CKM stage 4 | T1 (low TyG) | 8.08 | 0.7 (-1.2, 2.7) | 1.0 (-0.6, 2.5) | 0.5 | 0.2 |
| fasting_only | All-cause death | Hypotension SAE | CKM stage 4 | T2 (middle TyG) | 8.56 | 1.8 (0.3, 3.3) | 1.6 (0.4, 2.7) | 0.5 | 1.0 |
| fasting_only | All-cause death | Hypotension SAE | CKM stage 4 | T3 (high TyG) | 9.11 | 3.1 (0.7, 5.3) | 2.2 (0.4, 4.0) | 0.5 | 2.0 |
| fasting_only | All-cause death | Hypotension SAE | CKM stage 2 | T1 (low TyG) | 8.08 | 0.1 (-0.7, 1.0) | 0.6 (-0.3, 1.6) | 1.0 | -0.5 |
| fasting_only | All-cause death | Hypotension SAE | CKM stage 2 | T2 (middle TyG) | 8.56 | 0.2 (-0.4, 0.9) | 0.4 (-0.2, 1.0) | 1.0 | -0.2 |
| fasting_only | All-cause death | Hypotension SAE | CKM stage 2 | T3 (high TyG) | 9.11 | 0.3 (-0.6, 1.1) | 0.3 (-0.5, 1.1) | 1.0 | 0.0 |
| fasting_only | All-cause death | Hypotension SAE | CKM stage 3 | T1 (low TyG) | 8.08 | -1.0 (-2.9, 0.7) | 0.8 (-1.0, 2.5) | 1.0 | -1.8 |
| fasting_only | All-cause death | Hypotension SAE | CKM stage 3 | T2 (middle TyG) | 8.56 | 0.1 (-1.4, 1.7) | 0.8 (-0.5, 1.9) | 1.0 | -0.7 |
| fasting_only | All-cause death | Hypotension SAE | CKM stage 3 | T3 (high TyG) | 9.11 | 1.4 (-0.9, 3.5) | 0.8 (-0.9, 2.3) | 1.0 | 0.7 |
| fasting_only | All-cause death | Hypotension SAE | CKM stage 4 | T1 (low TyG) | 8.08 | 0.7 (-1.2, 2.7) | 1.0 (-0.6, 2.5) | 1.0 | -0.3 |
| fasting_only | All-cause death | Hypotension SAE | CKM stage 4 | T2 (middle TyG) | 8.56 | 1.8 (0.3, 3.3) | 1.6 (0.4, 2.7) | 1.0 | 0.2 |
| fasting_only | All-cause death | Hypotension SAE | CKM stage 4 | T3 (high TyG) | 9.11 | 3.1 (0.7, 5.3) | 2.2 (0.4, 4.0) | 1.0 | 0.9 |
| fasting_only | Primary composite outcome | Acute kidney injury SAE | CKM stage 2 | T1 (low TyG) | 8.08 | 1.1 (-0.0, 2.3) | 0.6 (-0.3, 1.7) | 0.5 | 0.8 |
| fasting_only | Primary composite outcome | Acute kidney injury SAE | CKM stage 2 | T2 (middle TyG) | 8.56 | 1.2 (0.3, 2.0) | 0.6 (-0.1, 1.3) | 0.5 | 0.9 |
| fasting_only | Primary composite outcome | Acute kidney injury SAE | CKM stage 2 | T3 (high TyG) | 9.11 | 1.2 (-0.1, 2.6) | 0.6 (-0.5, 1.6) | 0.5 | 0.9 |
| fasting_only | Primary composite outcome | Acute kidney injury SAE | CKM stage 3 | T1 (low TyG) | 8.08 | 0.2 (-2.2, 2.4) | 2.6 (0.4, 5.2) | 0.5 | -1.0 |
| fasting_only | Primary composite outcome | Acute kidney injury SAE | CKM stage 3 | T2 (middle TyG) | 8.56 | 1.2 (-0.7, 3.0) | 2.5 (0.8, 4.0) | 0.5 | -0.0 |
| fasting_only | Primary composite outcome | Acute kidney injury SAE | CKM stage 3 | T3 (high TyG) | 9.11 | 2.6 (-0.1, 5.1) | 2.2 (-0.6, 4.9) | 0.5 | 1.5 |
| fasting_only | Primary composite outcome | Acute kidney injury SAE | CKM stage 4 | T1 (low TyG) | 8.08 | 2.0 (-0.7, 4.7) | 1.0 (-1.4, 3.3) | 0.5 | 1.5 |
| fasting_only | Primary composite outcome | Acute kidney injury SAE | CKM stage 4 | T2 (middle TyG) | 8.56 | 2.4 (0.4, 4.4) | 1.5 (-0.1, 3.0) | 0.5 | 1.7 |
| fasting_only | Primary composite outcome | Acute kidney injury SAE | CKM stage 4 | T3 (high TyG) | 9.11 | 2.9 (-0.2, 6.3) | 2.0 (-0.1, 4.1) | 0.5 | 2.0 |
| fasting_only | Primary composite outcome | Acute kidney injury SAE | CKM stage 2 | T1 (low TyG) | 8.08 | 1.1 (-0.0, 2.3) | 0.6 (-0.3, 1.7) | 1.0 | 0.5 |
| fasting_only | Primary composite outcome | Acute kidney injury SAE | CKM stage 2 | T2 (middle TyG) | 8.56 | 1.2 (0.3, 2.0) | 0.6 (-0.1, 1.3) | 1.0 | 0.6 |
| fasting_only | Primary composite outcome | Acute kidney injury SAE | CKM stage 2 | T3 (high TyG) | 9.11 | 1.2 (-0.1, 2.6) | 0.6 (-0.5, 1.6) | 1.0 | 0.7 |
| fasting_only | Primary composite outcome | Acute kidney injury SAE | CKM stage 3 | T1 (low TyG) | 8.08 | 0.2 (-2.2, 2.4) | 2.6 (0.4, 5.2) | 1.0 | -2.3 |
| fasting_only | Primary composite outcome | Acute kidney injury SAE | CKM stage 3 | T2 (middle TyG) | 8.56 | 1.2 (-0.7, 3.0) | 2.5 (0.8, 4.0) | 1.0 | -1.3 |
| fasting_only | Primary composite outcome | Acute kidney injury SAE | CKM stage 3 | T3 (high TyG) | 9.11 | 2.6 (-0.1, 5.1) | 2.2 (-0.6, 4.9) | 1.0 | 0.4 |
| fasting_only | Primary composite outcome | Acute kidney injury SAE | CKM stage 4 | T1 (low TyG) | 8.08 | 2.0 (-0.7, 4.7) | 1.0 (-1.4, 3.3) | 1.0 | 1.0 |
| fasting_only | Primary composite outcome | Acute kidney injury SAE | CKM stage 4 | T2 (middle TyG) | 8.56 | 2.4 (0.4, 4.4) | 1.5 (-0.1, 3.0) | 1.0 | 0.9 |
| fasting_only | Primary composite outcome | Acute kidney injury SAE | CKM stage 4 | T3 (high TyG) | 9.11 | 2.9 (-0.2, 6.3) | 2.0 (-0.1, 4.1) | 1.0 | 1.0 |
| fasting_only | Primary composite outcome | Hypotension SAE | CKM stage 2 | T1 (low TyG) | 8.08 | 1.1 (-0.0, 2.3) | 0.6 (-0.3, 1.6) | 0.5 | 0.8 |
| fasting_only | Primary composite outcome | Hypotension SAE | CKM stage 2 | T2 (middle TyG) | 8.56 | 1.2 (0.3, 2.0) | 0.4 (-0.2, 1.0) | 0.5 | 1.0 |
| fasting_only | Primary composite outcome | Hypotension SAE | CKM stage 2 | T3 (high TyG) | 9.11 | 1.2 (-0.1, 2.6) | 0.3 (-0.5, 1.1) | 0.5 | 1.1 |
| fasting_only | Primary composite outcome | Hypotension SAE | CKM stage 3 | T1 (low TyG) | 8.08 | 0.2 (-2.2, 2.4) | 0.8 (-1.0, 2.5) | 0.5 | -0.2 |
| fasting_only | Primary composite outcome | Hypotension SAE | CKM stage 3 | T2 (middle TyG) | 8.56 | 1.2 (-0.7, 3.0) | 0.8 (-0.5, 1.9) | 0.5 | 0.8 |
| fasting_only | Primary composite outcome | Hypotension SAE | CKM stage 3 | T3 (high TyG) | 9.11 | 2.6 (-0.1, 5.1) | 0.8 (-0.9, 2.3) | 0.5 | 2.2 |
| fasting_only | Primary composite outcome | Hypotension SAE | CKM stage 4 | T1 (low TyG) | 8.08 | 2.0 (-0.7, 4.7) | 1.0 (-0.6, 2.5) | 0.5 | 1.5 |
| fasting_only | Primary composite outcome | Hypotension SAE | CKM stage 4 | T2 (middle TyG) | 8.56 | 2.4 (0.4, 4.4) | 1.6 (0.4, 2.7) | 0.5 | 1.6 |
| fasting_only | Primary composite outcome | Hypotension SAE | CKM stage 4 | T3 (high TyG) | 9.11 | 2.9 (-0.2, 6.3) | 2.2 (0.4, 4.0) | 0.5 | 1.8 |
| fasting_only | Primary composite outcome | Hypotension SAE | CKM stage 2 | T1 (low TyG) | 8.08 | 1.1 (-0.0, 2.3) | 0.6 (-0.3, 1.6) | 1.0 | 0.6 |
| fasting_only | Primary composite outcome | Hypotension SAE | CKM stage 2 | T2 (middle TyG) | 8.56 | 1.2 (0.3, 2.0) | 0.4 (-0.2, 1.0) | 1.0 | 0.7 |
| fasting_only | Primary composite outcome | Hypotension SAE | CKM stage 2 | T3 (high TyG) | 9.11 | 1.2 (-0.1, 2.6) | 0.3 (-0.5, 1.1) | 1.0 | 0.9 |
| fasting_only | Primary composite outcome | Hypotension SAE | CKM stage 3 | T1 (low TyG) | 8.08 | 0.2 (-2.2, 2.4) | 0.8 (-1.0, 2.5) | 1.0 | -0.5 |
| fasting_only | Primary composite outcome | Hypotension SAE | CKM stage 3 | T2 (middle TyG) | 8.56 | 1.2 (-0.7, 3.0) | 0.8 (-0.5, 1.9) | 1.0 | 0.4 |
| fasting_only | Primary composite outcome | Hypotension SAE | CKM stage 3 | T3 (high TyG) | 9.11 | 2.6 (-0.1, 5.1) | 0.8 (-0.9, 2.3) | 1.0 | 1.8 |
| fasting_only | Primary composite outcome | Hypotension SAE | CKM stage 4 | T1 (low TyG) | 8.08 | 2.0 (-0.7, 4.7) | 1.0 (-0.6, 2.5) | 1.0 | 1.0 |
| fasting_only | Primary composite outcome | Hypotension SAE | CKM stage 4 | T2 (middle TyG) | 8.56 | 2.4 (0.4, 4.4) | 1.6 (0.4, 2.7) | 1.0 | 0.8 |
| fasting_only | Primary composite outcome | Hypotension SAE | CKM stage 4 | T3 (high TyG) | 9.11 | 2.9 (-0.2, 6.3) | 2.2 (0.4, 4.0) | 1.0 | 0.7 |
| main | All-cause death | Acute kidney injury SAE | CKM stage 2 | T1 (low TyG) | 8.08 | 0.0 (-0.9, 1.0) | 0.6 (-0.4, 1.7) | 0.5 | -0.3 |
| main | All-cause death | Acute kidney injury SAE | CKM stage 2 | T2 (middle TyG) | 8.56 | 0.2 (-0.4, 0.8) | 0.6 (-0.1, 1.4) | 0.5 | -0.2 |
| main | All-cause death | Acute kidney injury SAE | CKM stage 2 | T3 (high TyG) | 9.11 | 0.3 (-0.5, 1.2) | 0.6 (-0.3, 1.6) | 0.5 | -0.0 |
| main | All-cause death | Acute kidney injury SAE | CKM stage 3 | T1 (low TyG) | 8.08 | -1.3 (-3.1, 0.9) | 2.9 (0.5, 5.6) | 0.5 | -2.7 |
| main | All-cause death | Acute kidney injury SAE | CKM stage 3 | T2 (middle TyG) | 8.56 | -0.2 (-1.5, 1.4) | 2.6 (1.0, 4.2) | 0.5 | -1.5 |
| main | All-cause death | Acute kidney injury SAE | CKM stage 3 | T3 (high TyG) | 9.11 | 1.2 (-0.9, 3.4) | 2.2 (-0.2, 4.6) | 0.5 | 0.1 |
| main | All-cause death | Acute kidney injury SAE | CKM stage 4 | T1 (low TyG) | 8.08 | 0.7 (-1.1, 2.6) | 1.6 (-0.8, 3.6) | 0.5 | -0.1 |
| main | All-cause death | Acute kidney injury SAE | CKM stage 4 | T2 (middle TyG) | 8.56 | 1.9 (0.5, 3.6) | 1.7 (0.2, 3.2) | 0.5 | 1.1 |
| main | All-cause death | Acute kidney injury SAE | CKM stage 4 | T3 (high TyG) | 9.11 | 3.4 (1.3, 5.6) | 1.7 (-0.3, 3.8) | 0.5 | 2.5 |
| main | All-cause death | Acute kidney injury SAE | CKM stage 2 | T1 (low TyG) | 8.08 | 0.0 (-0.9, 1.0) | 0.6 (-0.4, 1.7) | 1.0 | -0.6 |
| main | All-cause death | Acute kidney injury SAE | CKM stage 2 | T2 (middle TyG) | 8.56 | 0.2 (-0.4, 0.8) | 0.6 (-0.1, 1.4) | 1.0 | -0.5 |
| main | All-cause death | Acute kidney injury SAE | CKM stage 2 | T3 (high TyG) | 9.11 | 0.3 (-0.5, 1.2) | 0.6 (-0.3, 1.6) | 1.0 | -0.3 |
| main | All-cause death | Acute kidney injury SAE | CKM stage 3 | T1 (low TyG) | 8.08 | -1.3 (-3.1, 0.9) | 2.9 (0.5, 5.6) | 1.0 | -4.2 |
| main | All-cause death | Acute kidney injury SAE | CKM stage 3 | T2 (middle TyG) | 8.56 | -0.2 (-1.5, 1.4) | 2.6 (1.0, 4.2) | 1.0 | -2.8 |
| main | All-cause death | Acute kidney injury SAE | CKM stage 3 | T3 (high TyG) | 9.11 | 1.2 (-0.9, 3.4) | 2.2 (-0.2, 4.6) | 1.0 | -0.9 |
| main | All-cause death | Acute kidney injury SAE | CKM stage 4 | T1 (low TyG) | 8.08 | 0.7 (-1.1, 2.6) | 1.6 (-0.8, 3.6) | 1.0 | -0.8 |
| main | All-cause death | Acute kidney injury SAE | CKM stage 4 | T2 (middle TyG) | 8.56 | 1.9 (0.5, 3.6) | 1.7 (0.2, 3.2) | 1.0 | 0.3 |
| main | All-cause death | Acute kidney injury SAE | CKM stage 4 | T3 (high TyG) | 9.11 | 3.4 (1.3, 5.6) | 1.7 (-0.3, 3.8) | 1.0 | 1.6 |
| main | All-cause death | Hypotension SAE | CKM stage 2 | T1 (low TyG) | 8.08 | 0.0 (-0.9, 1.0) | 0.6 (-0.3, 1.5) | 0.5 | -0.2 |
| main | All-cause death | Hypotension SAE | CKM stage 2 | T2 (middle TyG) | 8.56 | 0.2 (-0.4, 0.8) | 0.4 (-0.2, 1.1) | 0.5 | -0.1 |
| main | All-cause death | Hypotension SAE | CKM stage 2 | T3 (high TyG) | 9.11 | 0.3 (-0.5, 1.2) | 0.2 (-0.6, 1.2) | 0.5 | 0.2 |
| main | All-cause death | Hypotension SAE | CKM stage 3 | T1 (low TyG) | 8.08 | -1.3 (-3.1, 0.9) | 0.8 (-1.3, 2.4) | 0.5 | -1.6 |
| main | All-cause death | Hypotension SAE | CKM stage 3 | T2 (middle TyG) | 8.56 | -0.2 (-1.5, 1.4) | 0.7 (-0.5, 1.8) | 0.5 | -0.5 |
| main | All-cause death | Hypotension SAE | CKM stage 3 | T3 (high TyG) | 9.11 | 1.2 (-0.9, 3.4) | 0.7 (-0.9, 2.0) | 0.5 | 0.9 |
| main | All-cause death | Hypotension SAE | CKM stage 4 | T1 (low TyG) | 8.08 | 0.7 (-1.1, 2.6) | 1.1 (-0.5, 2.8) | 0.5 | 0.2 |
| main | All-cause death | Hypotension SAE | CKM stage 4 | T2 (middle TyG) | 8.56 | 1.9 (0.5, 3.6) | 1.8 (0.6, 3.0) | 0.5 | 1.0 |
| main | All-cause death | Hypotension SAE | CKM stage 4 | T3 (high TyG) | 9.11 | 3.4 (1.3, 5.6) | 2.4 (0.7, 4.2) | 0.5 | 2.1 |
| main | All-cause death | Hypotension SAE | CKM stage 2 | T1 (low TyG) | 8.08 | 0.0 (-0.9, 1.0) | 0.6 (-0.3, 1.5) | 1.0 | -0.5 |
| main | All-cause death | Hypotension SAE | CKM stage 2 | T2 (middle TyG) | 8.56 | 0.2 (-0.4, 0.8) | 0.4 (-0.2, 1.1) | 1.0 | -0.3 |
| main | All-cause death | Hypotension SAE | CKM stage 2 | T3 (high TyG) | 9.11 | 0.3 (-0.5, 1.2) | 0.2 (-0.6, 1.2) | 1.0 | 0.0 |
| main | All-cause death | Hypotension SAE | CKM stage 3 | T1 (low TyG) | 8.08 | -1.3 (-3.1, 0.9) | 0.8 (-1.3, 2.4) | 1.0 | -2.0 |
| main | All-cause death | Hypotension SAE | CKM stage 3 | T2 (middle TyG) | 8.56 | -0.2 (-1.5, 1.4) | 0.7 (-0.5, 1.8) | 1.0 | -0.9 |
| main | All-cause death | Hypotension SAE | CKM stage 3 | T3 (high TyG) | 9.11 | 1.2 (-0.9, 3.4) | 0.7 (-0.9, 2.0) | 1.0 | 0.5 |
| main | All-cause death | Hypotension SAE | CKM stage 4 | T1 (low TyG) | 8.08 | 0.7 (-1.1, 2.6) | 1.1 (-0.5, 2.8) | 1.0 | -0.4 |
| main | All-cause death | Hypotension SAE | CKM stage 4 | T2 (middle TyG) | 8.56 | 1.9 (0.5, 3.6) | 1.8 (0.6, 3.0) | 1.0 | 0.2 |
| main | All-cause death | Hypotension SAE | CKM stage 4 | T3 (high TyG) | 9.11 | 3.4 (1.3, 5.6) | 2.4 (0.7, 4.2) | 1.0 | 0.9 |
| main | Primary composite outcome | Acute kidney injury SAE | CKM stage 2 | T1 (low TyG) | 8.08 | 1.1 (-0.0, 2.2) | 0.6 (-0.4, 1.7) | 0.5 | 0.8 |
| main | Primary composite outcome | Acute kidney injury SAE | CKM stage 2 | T2 (middle TyG) | 8.56 | 1.2 (0.4, 2.1) | 0.6 (-0.1, 1.4) | 0.5 | 0.9 |
| main | Primary composite outcome | Acute kidney injury SAE | CKM stage 2 | T3 (high TyG) | 9.11 | 1.4 (0.2, 2.7) | 0.6 (-0.3, 1.6) | 0.5 | 1.1 |
| main | Primary composite outcome | Acute kidney injury SAE | CKM stage 3 | T1 (low TyG) | 8.08 | -0.3 (-2.8, 2.0) | 2.9 (0.5, 5.6) | 0.5 | -1.8 |
| main | Primary composite outcome | Acute kidney injury SAE | CKM stage 3 | T2 (middle TyG) | 8.56 | 1.1 (-0.7, 3.1) | 2.6 (1.0, 4.2) | 0.5 | -0.2 |
| main | Primary composite outcome | Acute kidney injury SAE | CKM stage 3 | T3 (high TyG) | 9.11 | 3.2 (0.6, 5.9) | 2.2 (-0.2, 4.6) | 0.5 | 2.1 |
| main | Primary composite outcome | Acute kidney injury SAE | CKM stage 4 | T1 (low TyG) | 8.08 | 2.6 (0.0, 5.3) | 1.6 (-0.8, 3.6) | 0.5 | 1.8 |
| main | Primary composite outcome | Acute kidney injury SAE | CKM stage 4 | T2 (middle TyG) | 8.56 | 2.8 (0.8, 4.8) | 1.7 (0.2, 3.2) | 0.5 | 2.0 |
| main | Primary composite outcome | Acute kidney injury SAE | CKM stage 4 | T3 (high TyG) | 9.11 | 3.0 (-0.4, 6.5) | 1.7 (-0.3, 3.8) | 0.5 | 2.2 |
| main | Primary composite outcome | Acute kidney injury SAE | CKM stage 2 | T1 (low TyG) | 8.08 | 1.1 (-0.0, 2.2) | 0.6 (-0.4, 1.7) | 1.0 | 0.5 |
| main | Primary composite outcome | Acute kidney injury SAE | CKM stage 2 | T2 (middle TyG) | 8.56 | 1.2 (0.4, 2.1) | 0.6 (-0.1, 1.4) | 1.0 | 0.6 |
| main | Primary composite outcome | Acute kidney injury SAE | CKM stage 2 | T3 (high TyG) | 9.11 | 1.4 (0.2, 2.7) | 0.6 (-0.3, 1.6) | 1.0 | 0.8 |
| main | Primary composite outcome | Acute kidney injury SAE | CKM stage 3 | T1 (low TyG) | 8.08 | -0.3 (-2.8, 2.0) | 2.9 (0.5, 5.6) | 1.0 | -3.3 |
| main | Primary composite outcome | Acute kidney injury SAE | CKM stage 3 | T2 (middle TyG) | 8.56 | 1.1 (-0.7, 3.1) | 2.6 (1.0, 4.2) | 1.0 | -1.5 |
| main | Primary composite outcome | Acute kidney injury SAE | CKM stage 3 | T3 (high TyG) | 9.11 | 3.2 (0.6, 5.9) | 2.2 (-0.2, 4.6) | 1.0 | 1.0 |
| main | Primary composite outcome | Acute kidney injury SAE | CKM stage 4 | T1 (low TyG) | 8.08 | 2.6 (0.0, 5.3) | 1.6 (-0.8, 3.6) | 1.0 | 1.1 |
| main | Primary composite outcome | Acute kidney injury SAE | CKM stage 4 | T2 (middle TyG) | 8.56 | 2.8 (0.8, 4.8) | 1.7 (0.2, 3.2) | 1.0 | 1.2 |
| main | Primary composite outcome | Acute kidney injury SAE | CKM stage 4 | T3 (high TyG) | 9.11 | 3.0 (-0.4, 6.5) | 1.7 (-0.3, 3.8) | 1.0 | 1.3 |
| main | Primary composite outcome | Hypotension SAE | CKM stage 2 | T1 (low TyG) | 8.08 | 1.1 (-0.0, 2.2) | 0.6 (-0.3, 1.5) | 0.5 | 0.8 |
| main | Primary composite outcome | Hypotension SAE | CKM stage 2 | T2 (middle TyG) | 8.56 | 1.2 (0.4, 2.1) | 0.4 (-0.2, 1.1) | 0.5 | 1.0 |
| main | Primary composite outcome | Hypotension SAE | CKM stage 2 | T3 (high TyG) | 9.11 | 1.4 (0.2, 2.7) | 0.2 (-0.6, 1.2) | 0.5 | 1.3 |
| main | Primary composite outcome | Hypotension SAE | CKM stage 3 | T1 (low TyG) | 8.08 | -0.3 (-2.8, 2.0) | 0.8 (-1.3, 2.4) | 0.5 | -0.7 |
| main | Primary composite outcome | Hypotension SAE | CKM stage 3 | T2 (middle TyG) | 8.56 | 1.1 (-0.7, 3.1) | 0.7 (-0.5, 1.8) | 0.5 | 0.7 |
| main | Primary composite outcome | Hypotension SAE | CKM stage 3 | T3 (high TyG) | 9.11 | 3.2 (0.6, 5.9) | 0.7 (-0.9, 2.0) | 0.5 | 2.8 |
| main | Primary composite outcome | Hypotension SAE | CKM stage 4 | T1 (low TyG) | 8.08 | 2.6 (0.0, 5.3) | 1.1 (-0.5, 2.8) | 0.5 | 2.1 |
| main | Primary composite outcome | Hypotension SAE | CKM stage 4 | T2 (middle TyG) | 8.56 | 2.8 (0.8, 4.8) | 1.8 (0.6, 3.0) | 0.5 | 1.9 |
| main | Primary composite outcome | Hypotension SAE | CKM stage 4 | T3 (high TyG) | 9.11 | 3.0 (-0.4, 6.5) | 2.4 (0.7, 4.2) | 0.5 | 1.8 |
| main | Primary composite outcome | Hypotension SAE | CKM stage 2 | T1 (low TyG) | 8.08 | 1.1 (-0.0, 2.2) | 0.6 (-0.3, 1.5) | 1.0 | 0.5 |
| main | Primary composite outcome | Hypotension SAE | CKM stage 2 | T2 (middle TyG) | 8.56 | 1.2 (0.4, 2.1) | 0.4 (-0.2, 1.1) | 1.0 | 0.8 |
| main | Primary composite outcome | Hypotension SAE | CKM stage 2 | T3 (high TyG) | 9.11 | 1.4 (0.2, 2.7) | 0.2 (-0.6, 1.2) | 1.0 | 1.2 |
| main | Primary composite outcome | Hypotension SAE | CKM stage 3 | T1 (low TyG) | 8.08 | -0.3 (-2.8, 2.0) | 0.8 (-1.3, 2.4) | 1.0 | -1.1 |
| main | Primary composite outcome | Hypotension SAE | CKM stage 3 | T2 (middle TyG) | 8.56 | 1.1 (-0.7, 3.1) | 0.7 (-0.5, 1.8) | 1.0 | 0.4 |
| main | Primary composite outcome | Hypotension SAE | CKM stage 3 | T3 (high TyG) | 9.11 | 3.2 (0.6, 5.9) | 0.7 (-0.9, 2.0) | 1.0 | 2.5 |
| main | Primary composite outcome | Hypotension SAE | CKM stage 4 | T1 (low TyG) | 8.08 | 2.6 (0.0, 5.3) | 1.1 (-0.5, 2.8) | 1.0 | 1.5 |
| main | Primary composite outcome | Hypotension SAE | CKM stage 4 | T2 (middle TyG) | 8.56 | 2.8 (0.8, 4.8) | 1.8 (0.6, 3.0) | 1.0 | 1.1 |
| main | Primary composite outcome | Hypotension SAE | CKM stage 4 | T3 (high TyG) | 9.11 | 3.0 (-0.4, 6.5) | 2.4 (0.7, 4.2) | 1.0 | 0.6 |

*Simplified net benefit = efficacy ARR - harm weight × harm increase. Harm weights are sensitivity parameters and not validated patient-centered utility weights. These analyses are exploratory.*

Abbreviations: ARR, absolute risk reduction; BP, blood pressure; CKD, chronic kidney disease; CKM, cardiovascular-kidney-metabolic; eGFR, estimated glomerular filtration rate; HDL, high-density lipoprotein; SAE, serious adverse event; SPRINT, Systolic Blood Pressure Intervention Trial; TyG, triglyceride-glucose index; UACR, urinary albumin-to-creatinine ratio.

***Supplementary Table S9. Side-by-side stratum-specific HRs, standardized 3-year risks, ARR, and NNT display rules.***

| **Cohort** | **Outcome** | **CKM stage** | **TyG tertile** | **TyG median** | **N in stratum** | **Events in stratum** | **Intensive BP treatment vs standard BP treatment HR (95% CI)** | **HR P value** | **3-year risk, standard BP/intensive BP (%)** | **ARR, percentage points (95% CI)** | **NNT** | **NNT display rule** | **HR estimation status** |
| --- | --- | --- | --- | --- | --- | --- | --- | --- | --- | --- | --- | --- | --- |
| Main analytic cohort | All-cause death | CKM stage 2 | T1 (low TyG) | 8.08 | 1198 | 25 | 1.24 (0.56, 2.73) | 0.5972 | 1.3 / 1.3 | 0.0 (-0.9, 1.0) | Not shown: ARR 95% CI crosses zero | Estimated | |
| Main analytic cohort | All-cause death | CKM stage 2 | T2 (middle TyG) | 8.56 | 1190 | 26 | 0.59 (0.27, 1.31) | 0.1964 | 1.4 / 1.2 | 0.2 (-0.4, 0.8) | Not shown: ARR 95% CI crosses zero | Estimated | |
| Main analytic cohort | All-cause death | CKM stage 2 | T3 (high TyG) | 9.11 | 1330 | 28 | 0.88 (0.42, 1.85) | 0.7320 | 1.4 / 1.2 | 0.3 (-0.5, 1.2) | Not shown: ARR 95% CI crosses zero | Estimated | |
| Main analytic cohort | All-cause death | CKM stage 3 | T1 (low TyG) | 8.08 | 592 | 36 | 1.13 (0.58, 2.21) | 0.7175 | 3.5 / 4.8 | -1.3 (-3.1, 0.9) | Not shown: ARR < 0 | Estimated | |
| Main analytic cohort | All-cause death | CKM stage 3 | T2 (middle TyG) | 8.56 | 603 | 44 | 1.16 (0.63, 2.11) | 0.6352 | 4.3 / 4.5 | -0.2 (-1.5, 1.4) | Not shown: ARR < 0 | Estimated | |
| Main analytic cohort | All-cause death | CKM stage 3 | T3 (high TyG) | 9.11 | 545 | 42 | 0.75 (0.41, 1.40) | 0.3693 | 5.3 / 4.1 | 1.2 (-0.9, 3.4) | Not shown: ARR 95% CI crosses zero | Estimated | |
| Main analytic cohort | All-cause death | CKM stage 4 | T1 (low TyG) | 8.08 | 889 | 78 | 0.92 (0.59, 1.43) | 0.7049 | 5.7 / 5.0 | 0.7 (-1.1, 2.6) | Not shown: ARR 95% CI crosses zero | Estimated | |
| Main analytic cohort | All-cause death | CKM stage 4 | T2 (middle TyG) | 8.56 | 885 | 71 | 0.60 (0.37, 0.96) | 0.0339 | 6.5 / 4.5 | 1.9 (0.5, 3.6) | 53 | Displayed | Estimated |
| Main analytic cohort | All-cause death | CKM stage 4 | T3 (high TyG) | 9.11 | 804 | 70 | 0.56 (0.34, 0.91) | 0.0193 | 7.4 / 4.1 | 3.4 (1.3, 5.6) | 30 | Displayed | Estimated |
| Main analytic cohort | Primary composite outcome | CKM stage 2 | T1 (low TyG) | 8.08 | 1198 | 32 | 0.86 (0.43, 1.72) | 0.6637 | 2.7 / 1.7 | 1.1 (-0.0, 2.2) | Not shown: ARR 95% CI crosses zero | Estimated | |
| Main analytic cohort | Primary composite outcome | CKM stage 2 | T2 (middle TyG) | 8.56 | 1190 | 43 | 0.33 (0.16, 0.65) | 0.0014 | 3.1 / 1.9 | 1.2 (0.4, 2.1) | 81 | Displayed | Estimated |
| Main analytic cohort | Primary composite outcome | CKM stage 2 | T3 (high TyG) | 9.11 | 1330 | 54 | 0.74 (0.43, 1.27) | 0.2769 | 3.6 / 2.2 | 1.4 (0.2, 2.7) | 71 | Displayed | Estimated |
| Main analytic cohort | Primary composite outcome | CKM stage 3 | T1 (low TyG) | 8.08 | 592 | 36 | 1.20 (0.62, 2.32) | 0.5882 | 4.9 / 5.3 | -0.3 (-2.8, 2.0) | Not shown: ARR < 0 | Estimated | |
| Main analytic cohort | Primary composite outcome | CKM stage 3 | T2 (middle TyG) | 8.56 | 603 | 58 | 0.68 (0.40, 1.15) | 0.1483 | 6.3 / 5.2 | 1.1 (-0.7, 3.1) | Not shown: ARR 95% CI crosses zero | Estimated | |
| Main analytic cohort | Primary composite outcome | CKM stage 3 | T3 (high TyG) | 9.11 | 545 | 40 | 0.67 (0.36, 1.26) | 0.2166 | 8.3 / 5.1 | 3.2 (0.6, 5.9) | 32 | Displayed | Estimated |
| Main analytic cohort | Primary composite outcome | CKM stage 4 | T1 (low TyG) | 8.08 | 889 | 106 | 0.76 (0.52, 1.12) | 0.1629 | 10.9 / 8.3 | 2.6 (0.0, 5.3) | 39 | Displayed | Estimated |
| Main analytic cohort | Primary composite outcome | CKM stage 4 | T2 (middle TyG) | 8.56 | 885 | 127 | 0.75 (0.53, 1.07) | 0.1138 | 12.6 / 9.8 | 2.8 (0.8, 4.8) | 36 | Displayed | Estimated |
| Main analytic cohort | Primary composite outcome | CKM stage 4 | T3 (high TyG) | 9.11 | 804 | 135 | 0.74 (0.53, 1.05) | 0.0891 | 14.7 / 11.7 | 3.0 (-0.4, 6.5) | Not shown: ARR 95% CI crosses zero | Estimated | |
| Fasting-only sensitivity cohort | All-cause death | CKM stage 2 | T1 (low TyG) | 8.08 | 1117 | 24 | 1.20 (0.53, 2.67) | 0.6634 | 1.4 / 1.3 | 0.1 (-0.7, 1.0) | Not shown: ARR 95% CI crosses zero | Estimated | |
| Fasting-only sensitivity cohort | All-cause death | CKM stage 2 | T2 (middle TyG) | 8.56 | 1125 | 26 | 0.56 (0.26, 1.25) | 0.1587 | 1.5 / 1.3 | 0.2 (-0.4, 0.9) | Not shown: ARR 95% CI crosses zero | Estimated | |
| Fasting-only sensitivity cohort | All-cause death | CKM stage 2 | T3 (high TyG) | 9.11 | 1254 | 28 | 0.87 (0.42, 1.83) | 0.7193 | 1.6 / 1.2 | 0.3 (-0.6, 1.1) | Not shown: ARR 95% CI crosses zero | Estimated | |
| Fasting-only sensitivity cohort | All-cause death | CKM stage 3 | T1 (low TyG) | 8.08 | 556 | 34 | 1.24 (0.62, 2.46) | 0.5376 | 3.7 / 4.7 | -1.0 (-2.9, 0.7) | Not shown: ARR < 0 | Estimated | |
| Fasting-only sensitivity cohort | All-cause death | CKM stage 3 | T2 (middle TyG) | 8.56 | 558 | 40 | 0.95 (0.50, 1.78) | 0.8620 | 4.3 / 4.2 | 0.1 (-1.4, 1.7) | Not shown: ARR 95% CI crosses zero | Estimated | |
| Fasting-only sensitivity cohort | All-cause death | CKM stage 3 | T3 (high TyG) | 9.11 | 509 | 37 | 0.79 (0.41, 1.52) | 0.4873 | 5.1 / 3.7 | 1.4 (-0.9, 3.5) | Not shown: ARR 95% CI crosses zero | Estimated | |
| Fasting-only sensitivity cohort | All-cause death | CKM stage 4 | T1 (low TyG) | 8.08 | 837 | 69 | 0.90 (0.56, 1.44) | 0.6617 | 5.4 / 4.7 | 0.7 (-1.2, 2.7) | Not shown: ARR 95% CI crosses zero | Estimated | |
| Fasting-only sensitivity cohort | All-cause death | CKM stage 4 | T2 (middle TyG) | 8.56 | 824 | 63 | 0.64 (0.39, 1.07) | 0.0892 | 6.1 / 4.2 | 1.8 (0.3, 3.3) | 55 | Displayed | Estimated |
| Fasting-only sensitivity cohort | All-cause death | CKM stage 4 | T3 (high TyG) | 9.11 | 746 | 60 | 0.52 (0.30, 0.88) | 0.0158 | 6.9 / 3.7 | 3.1 (0.7, 5.3) | 32 | Displayed | Estimated |
| Fasting-only sensitivity cohort | Primary composite outcome | CKM stage 2 | T1 (low TyG) | 8.08 | 1117 | 32 | 0.88 (0.44, 1.77) | 0.7223 | 2.8 / 1.7 | 1.1 (-0.0, 2.3) | Not shown: ARR 95% CI crosses zero | Estimated | |
| Fasting-only sensitivity cohort | Primary composite outcome | CKM stage 2 | T2 (middle TyG) | 8.56 | 1125 | 40 | 0.31 (0.15, 0.63) | 0.0013 | 3.1 / 1.9 | 1.2 (0.3, 2.0) | 86 | Displayed | Estimated |
| Fasting-only sensitivity cohort | Primary composite outcome | CKM stage 2 | T3 (high TyG) | 9.11 | 1254 | 49 | 0.82 (0.47, 1.44) | 0.4846 | 3.4 / 2.2 | 1.2 (-0.1, 2.6) | Not shown: ARR 95% CI crosses zero | Estimated | |
| Fasting-only sensitivity cohort | Primary composite outcome | CKM stage 3 | T1 (low TyG) | 8.08 | 556 | 31 | 1.11 (0.54, 2.26) | 0.7778 | 4.9 / 4.7 | 0.2 (-2.2, 2.4) | Not shown: ARR 95% CI crosses zero | Estimated | |
| Fasting-only sensitivity cohort | Primary composite outcome | CKM stage 3 | T2 (middle TyG) | 8.56 | 558 | 52 | 0.65 (0.37, 1.14) | 0.1342 | 6.1 / 4.9 | 1.2 (-0.7, 3.0) | Not shown: ARR 95% CI crosses zero | Estimated | |
| Fasting-only sensitivity cohort | Primary composite outcome | CKM stage 3 | T3 (high TyG) | 9.11 | 509 | 38 | 0.75 (0.40, 1.44) | 0.3892 | 7.8 / 5.2 | 2.6 (-0.1, 5.1) | Not shown: ARR 95% CI crosses zero | Estimated | |
| Fasting-only sensitivity cohort | Primary composite outcome | CKM stage 4 | T1 (low TyG) | 8.08 | 837 | 96 | 0.80 (0.53, 1.19) | 0.2738 | 10.3 / 8.3 | 2.0 (-0.7, 4.7) | Not shown: ARR 95% CI crosses zero | Estimated | |
| Fasting-only sensitivity cohort | Primary composite outcome | CKM stage 4 | T2 (middle TyG) | 8.56 | 824 | 118 | 0.80 (0.55, 1.15) | 0.2240 | 11.9 / 9.5 | 2.4 (0.4, 4.4) | 42 | Displayed | Estimated |
| Fasting-only sensitivity cohort | Primary composite outcome | CKM stage 4 | T3 (high TyG) | 9.11 | 746 | 120 | 0.73 (0.51, 1.05) | 0.0885 | 14.1 / 11.1 | 2.9 (-0.2, 6.3) | Not shown: ARR 95% CI crosses zero | Estimated | |

*Stratum-specific HRs compare randomized intensive BP treatment with standard BP treatment within each CKM stage and TyG tertile. Standardized 3-year risks were estimated under counterfactual standard and intensive BP treatment strategies using Cox-standardized survival prediction. ARR was defined as standardized risk under standard BP treatment minus standardized risk under intensive BP treatment. NNT was displayed only when ARR was positive, the 95% CI excluded zero, and ARR was at least 0.5 percentage points; otherwise, NNT was not shown.*

***Supplementary Table S10. Expanded-adjustment complete-case sensitivity analyses.***

**Panel A. Main treatment HRs: parsimonious versus expanded adjustment.**

| **Cohort** | **Outcome** | **Model** | **N in model** | **Events in model** | **HR (95% CI)** | **P value** | **Note** |
| --- | --- | --- | --- | --- | --- | --- | --- |
| Main analytic cohort | All-cause death | Parsimonious primary model | 5639 | 199 | 0.81 (0.61, 1.07) | 0.1400 | Primary model |
| Main analytic cohort | All-cause death | Expanded adjustment sensitivity model | 5639 | 199 | 0.81 (0.61, 1.08) | 0.1470 | Expanded sensitivity model |
| Main analytic cohort | Primary composite outcome | Parsimonious primary model | 5639 | 351 | 0.77 (0.63, 0.96) | 0.0174 | Primary model |
| Main analytic cohort | Primary composite outcome | Expanded adjustment sensitivity model | 5639 | 351 | 0.78 (0.63, 0.97) | 0.0221 | Expanded sensitivity model |
| Fasting-only sensitivity cohort | All-cause death | Parsimonious primary model | 5298 | 189 | 0.81 (0.61, 1.09) | 0.1620 | Primary model |
| Fasting-only sensitivity cohort | All-cause death | Expanded adjustment sensitivity model | 5298 | 189 | 0.84 (0.63, 1.12) | 0.2390 | Expanded sensitivity model |
| Fasting-only sensitivity cohort | Primary composite outcome | Parsimonious primary model | 5298 | 330 | 0.80 (0.65, 1.00) | 0.0475 | Primary model |
| Fasting-only sensitivity cohort | Primary composite outcome | Expanded adjustment sensitivity model | 5298 | 330 | 0.81 (0.65, 1.01) | 0.0612 | Expanded sensitivity model |

*Expanded models included race/ethnicity indicators, BMI, current smoking, baseline BP medication count, total cholesterol, HDL cholesterol, LDL cholesterol, UACR, and aspirin use, in addition to the primary model covariates.*

**Panel B. Formal interaction tests under expanded adjustment sensitivity model.**

| **Cohort** | **Outcome** | **N in model** | **Events in model** | **Interaction test** | **Chi-square** | **df** | **P value** | **Interpretation** |
| --- | --- | --- | --- | --- | --- | --- | --- | --- |
| Main analytic cohort | All-cause death | 5639 | 199 | Intensive BP treatment x CKM stage | 0.35 | 2 | 0.8400 | No nominal evidence of interaction |
| Main analytic cohort | All-cause death | 5639 | 199 | Intensive BP treatment x TyG | 2.702 | 1 | 0.1000 | No nominal evidence of interaction |
| Main analytic cohort | All-cause death | 5639 | 199 | Intensive BP treatment x CKM stage x TyG | 2.096 | 2 | 0.3510 | No nominal evidence of interaction |
| Main analytic cohort | All-cause death | 5639 | 199 | Any intensive-treatment effect modification by CKM stage or TyG | 3.325 | 5 | 0.6500 | No nominal evidence of interaction |
| Main analytic cohort | Primary composite outcome | 5639 | 351 | Intensive BP treatment x CKM stage | 6.276 | 2 | 0.0434 | Nominal evidence of interaction |
| Main analytic cohort | Primary composite outcome | 5639 | 351 | Intensive BP treatment x TyG | 0.098 | 1 | 0.7542 | No nominal evidence of interaction |
| Main analytic cohort | Primary composite outcome | 5639 | 351 | Intensive BP treatment x CKM stage x TyG | 1.599 | 2 | 0.4495 | No nominal evidence of interaction |
| Main analytic cohort | Primary composite outcome | 5639 | 351 | Any intensive-treatment effect modification by CKM stage or TyG | 6.802 | 5 | 0.2358 | No nominal evidence of interaction |
| Fasting-only sensitivity cohort | All-cause death | 5298 | 189 | Intensive BP treatment x CKM stage | 0.361 | 2 | 0.8350 | No nominal evidence of interaction |
| Fasting-only sensitivity cohort | All-cause death | 5298 | 189 | Intensive BP treatment x TyG | 2.654 | 1 | 0.1030 | No nominal evidence of interaction |
| Fasting-only sensitivity cohort | All-cause death | 5298 | 189 | Intensive BP treatment x CKM stage x TyG | 1.163 | 2 | 0.5590 | No nominal evidence of interaction |
| Fasting-only sensitivity cohort | All-cause death | 5298 | 189 | Any intensive-treatment effect modification by CKM stage or TyG | 3.081 | 5 | 0.6880 | No nominal evidence of interaction |
| Fasting-only sensitivity cohort | Primary composite outcome | 5298 | 330 | Intensive BP treatment x CKM stage | 7.201 | 2 | 0.0273 | Nominal evidence of interaction |
| Fasting-only sensitivity cohort | Primary composite outcome | 5298 | 330 | Intensive BP treatment x TyG | 0.08 | 1 | 0.7770 | No nominal evidence of interaction |
| Fasting-only sensitivity cohort | Primary composite outcome | 5298 | 330 | Intensive BP treatment x CKM stage x TyG | 0.181 | 2 | 0.9136 | No nominal evidence of interaction |
| Fasting-only sensitivity cohort | Primary composite outcome | 5298 | 330 | Any intensive-treatment effect modification by CKM stage or TyG | 7.695 | 5 | 0.1738 | No nominal evidence of interaction |

**Panel C. Expanded-adjustment Cox-standardized 3-year ARR estimates.**

| **Cohort** | **Outcome** | **CKM stage** | **TyG tertile** | **TyG median** | **N in model** | **Events in model** | **3-year risk, standard BP/intensive BP (%)** | **ARR, percentage points (95% CI)** | **NNT** | **NNT display rule** | **Bootstrap successful** |
| --- | --- | --- | --- | --- | --- | --- | --- | --- | --- | --- | --- |
| Main analytic cohort | All-cause death | CKM stage 2 | T1 (low TyG) | 8.1 | 5639 | 199 | 1.8 / 1.6 | 0.2 (-0.8, 1.4) | Not shown: ARR CI crosses zero | 300 |  |
| Main analytic cohort | All-cause death | CKM stage 2 | T2 (middle TyG) | 8.59 | 5639 | 199 | 1.7 / 1.4 | 0.3 (-0.4, 1.1) | Not shown: ARR CI crosses zero | 300 |  |
| Main analytic cohort | All-cause death | CKM stage 2 | T3 (high TyG) | 9.13 | 5639 | 199 | 1.5 / 1.2 | 0.3 (-0.6, 1.4) | Not shown: ARR CI crosses zero | 300 |  |
| Main analytic cohort | All-cause death | CKM stage 3 | T1 (low TyG) | 8.1 | 5639 | 199 | 1.9 / 3.3 | -1.4 (-3.7, 0.9) | Not shown: ARR not positive | 300 |  |
| Main analytic cohort | All-cause death | CKM stage 3 | T2 (middle TyG) | 8.59 | 5639 | 199 | 2.7 / 2.7 | -0.1 (-1.8, 1.4) | Not shown: ARR not positive | 300 |  |
| Main analytic cohort | All-cause death | CKM stage 3 | T3 (high TyG) | 9.13 | 5639 | 199 | 3.9 / 2.2 | 1.7 (-0.8, 4.5) | Not shown: ARR CI crosses zero | 300 |  |
| Main analytic cohort | All-cause death | CKM stage 4 | T1 (low TyG) | 8.1 | 5639 | 199 | 3.6 / 3.2 | 0.4 (-1.8, 2.2) | Not shown: ARR CI crosses zero | 300 |  |
| Main analytic cohort | All-cause death | CKM stage 4 | T2 (middle TyG) | 8.59 | 5639 | 199 | 3.9 / 2.9 | 0.9 (-0.5, 2.3) | Not shown: ARR CI crosses zero | 300 |  |
| Main analytic cohort | All-cause death | CKM stage 4 | T3 (high TyG) | 9.13 | 5639 | 199 | 4.3 / 2.7 | 1.6 (-0.4, 3.9) | Not shown: ARR CI crosses zero | 300 |  |
| Main analytic cohort | Primary composite outcome | CKM stage 2 | T1 (low TyG) | 8.1 | 5639 | 351 | 2.8 / 1.5 | 1.2 (0.1, 2.3) | 81 | Displayed | 300 |
| Main analytic cohort | Primary composite outcome | CKM stage 2 | T2 (middle TyG) | 8.59 | 5639 | 351 | 3.6 / 1.9 | 1.6 (0.6, 2.6) | 61 | Displayed | 300 |
| Main analytic cohort | Primary composite outcome | CKM stage 2 | T3 (high TyG) | 9.13 | 5639 | 351 | 4.7 / 2.5 | 2.2 (0.8, 4.2) | 45 | Displayed | 300 |
| Main analytic cohort | Primary composite outcome | CKM stage 3 | T1 (low TyG) | 8.1 | 5639 | 351 | 2.3 / 3.8 | -1.4 (-3.8, 1.0) | Not shown: ARR not positive | 300 |  |
| Main analytic cohort | Primary composite outcome | CKM stage 3 | T2 (middle TyG) | 8.59 | 5639 | 351 | 4.0 / 4.6 | -0.7 (-2.8, 1.7) | Not shown: ARR not positive | 300 |  |
| Main analytic cohort | Primary composite outcome | CKM stage 3 | T3 (high TyG) | 9.13 | 5639 | 351 | 7.2 / 5.8 | 1.3 (-3.0, 5.7) | Not shown: ARR CI crosses zero | 300 |  |
| Main analytic cohort | Primary composite outcome | CKM stage 4 | T1 (low TyG) | 8.1 | 5639 | 351 | 7.1 / 6.2 | 0.9 (-1.8, 3.7) | Not shown: ARR CI crosses zero | 300 |  |
| Main analytic cohort | Primary composite outcome | CKM stage 4 | T2 (middle TyG) | 8.59 | 5639 | 351 | 9.3 / 8.4 | 0.9 (-1.5, 3.4) | Not shown: ARR CI crosses zero | 300 |  |
| Main analytic cohort | Primary composite outcome | CKM stage 4 | T3 (high TyG) | 9.13 | 5639 | 351 | 12.5 / 11.7 | 0.7 (-4.0, 5.4) | Not shown: ARR CI crosses zero | 300 |  |
| Fasting-only sensitivity cohort | All-cause death | CKM stage 2 | T1 (low TyG) | 8.1 | 5298 | 189 | 1.9 / 1.7 | 0.2 (-0.7, 1.3) | Not shown: ARR CI crosses zero | 300 |  |
| Fasting-only sensitivity cohort | All-cause death | CKM stage 2 | T2 (middle TyG) | 8.59 | 5298 | 189 | 1.8 / 1.5 | 0.3 (-0.5, 1.1) | Not shown: ARR CI crosses zero | 300 |  |
| Fasting-only sensitivity cohort | All-cause death | CKM stage 2 | T3 (high TyG) | 9.13 | 5298 | 189 | 1.6 / 1.3 | 0.3 (-0.6, 1.5) | Not shown: ARR CI crosses zero | 300 |  |
| Fasting-only sensitivity cohort | All-cause death | CKM stage 3 | T1 (low TyG) | 8.1 | 5298 | 189 | 1.8 / 3.1 | -1.3 (-3.6, 0.6) | Not shown: ARR not positive | 300 |  |
| Fasting-only sensitivity cohort | All-cause death | CKM stage 3 | T2 (middle TyG) | 8.59 | 5298 | 189 | 2.5 / 2.8 | -0.2 (-2.1, 1.4) | Not shown: ARR not positive | 300 |  |
| Fasting-only sensitivity cohort | All-cause death | CKM stage 3 | T3 (high TyG) | 9.13 | 5298 | 189 | 3.7 / 2.4 | 1.3 (-1.4, 4.2) | Not shown: ARR CI crosses zero | 300 |  |
| Fasting-only sensitivity cohort | All-cause death | CKM stage 4 | T1 (low TyG) | 8.1 | 5298 | 189 | 3.2 / 3.2 | 0.0 (-1.9, 1.8) | Not shown: ARR CI crosses zero | 300 |  |
| Fasting-only sensitivity cohort | All-cause death | CKM stage 4 | T2 (middle TyG) | 8.59 | 5298 | 189 | 3.6 / 2.8 | 0.8 (-0.7, 2.2) | Not shown: ARR CI crosses zero | 300 |  |
| Fasting-only sensitivity cohort | All-cause death | CKM stage 4 | T3 (high TyG) | 9.13 | 5298 | 189 | 4.1 / 2.5 | 1.6 (-0.7, 3.9) | Not shown: ARR CI crosses zero | 300 |  |
| Fasting-only sensitivity cohort | Primary composite outcome | CKM stage 2 | T1 (low TyG) | 8.1 | 5298 | 330 | 2.7 / 1.5 | 1.2 (0.1, 2.4) | 83 | Displayed | 300 |
| Fasting-only sensitivity cohort | Primary composite outcome | CKM stage 2 | T2 (middle TyG) | 8.59 | 5298 | 330 | 3.5 / 1.9 | 1.6 (0.6, 2.7) | 64 | Displayed | 300 |
| Fasting-only sensitivity cohort | Primary composite outcome | CKM stage 2 | T3 (high TyG) | 9.13 | 5298 | 330 | 4.6 / 2.4 | 2.1 (0.5, 4.2) | 47 | Displayed | 300 |
| Fasting-only sensitivity cohort | Primary composite outcome | CKM stage 3 | T1 (low TyG) | 8.1 | 5298 | 330 | 2.2 / 3.4 | -1.1 (-3.5, 1.3) | Not shown: ARR not positive | 300 |  |
| Fasting-only sensitivity cohort | Primary composite outcome | CKM stage 3 | T2 (middle TyG) | 8.59 | 5298 | 330 | 3.7 / 4.8 | -1.1 (-3.3, 1.7) | Not shown: ARR not positive | 300 |  |
| Fasting-only sensitivity cohort | Primary composite outcome | CKM stage 3 | T3 (high TyG) | 9.13 | 5298 | 330 | 6.5 / 7.1 | -0.6 (-6.0, 3.8) | Not shown: ARR not positive | 300 |  |
| Fasting-only sensitivity cohort | Primary composite outcome | CKM stage 4 | T1 (low TyG) | 8.1 | 5298 | 330 | 6.5 / 6.2 | 0.3 (-2.4, 2.8) | Not shown: ARR CI crosses zero | 300 |  |
| Fasting-only sensitivity cohort | Primary composite outcome | CKM stage 4 | T2 (middle TyG) | 8.59 | 5298 | 330 | 9.0 / 8.4 | 0.6 (-1.7, 3.1) | Not shown: ARR CI crosses zero | 300 |  |
| Fasting-only sensitivity cohort | Primary composite outcome | CKM stage 4 | T3 (high TyG) | 9.13 | 5298 | 330 | 13.0 / 11.8 | 1.2 (-3.7, 5.5) | Not shown: ARR CI crosses zero | 300 |  |
